# Supplementary material for: Bluebelle pilot randomised controlled trial of three wound dressing strategies to reduce surgical site infection in primary surgical wounds
Source: BMJ Open. 2020 Jan 12;10(1):e030615. doi: 10.1136/bmjopen-2019-030615 (PMC7045119; doi:10.1136/bmjopen-2019-030615)
Supplement: Author's manuscript [file bmjopen-2019-030615.draft_revisions.pdf]

BMJ Open

BMJ Open is committed to open peer review. As part of this commitment we make the peer review history of every article we publish publicly available.

When an article is published we post the peer reviewers' comments and the authors' responses online. We also post the versions of the paper that were used during peer review. These are the versions that the peer review comments apply to.

The versions of the paper that follow are the versions that were submitted during the peer review process. They are not the versions of record or the final published versions. They should not be cited or distributed as the published version of this manuscript.

BMJ Open is an open access journal and the full, final, typeset and author-corrected version of record of the manuscript is available on our site with no access controls, subscription charges or pay-per-view fees (http://bmjopen.bmj.com).

If you have any questions on BMJ Open's open peer review process please email info.bmjopen@bmj.com

BMJ Open

# The Bluebelle pilot randomised controlled trial of three wound dressing strategies to reduce surgical site infection in primary surgical wounds



| | |
|---|---|
| Journal: | *BMJ Open* |
| Manuscript ID | bmjopen-2019-030615 |
| Article Type: | Original research |
| Date Submitted by the Author: | 28-Mar-2019 |



| | |
|---|---|
| Complete List of Authors: | Blazeby, Jane; University of Bristol, Department of Population Health Sciences, Bristol Medical School |



| | |
|---|---|
| Keywords: | Randomised controlled trial, Pilot study, Surgical site infection, Wound dressing, Tissue adhesive as a dressing |


SCHOLARONE™
Manuscripts

# The Bluebelle pilot randomised controlled trial of three wound dressing strategies to reduce surgical site infection in primary surgical wounds

On behalf of the Bluebelle Study Group*

Author for correspondence:

Professor Jane M Blazeby

Centre of Surgical Research,

Bristol Medical School,

University of Bristol,

Canynge Hall,

39 Whatley Road,

Bristol BS8 2PS.

Tel: 0117 9287332

Email: j.m.blazeby@bristol.ac.uk

Word count: 2827

*Authors for 'Bluebelle Study Group':

1. Mark Woodward: University Hospitals Bristol NHS Foundation Trust, Bristol, UK
2. Nicky J Welton: Population Health Sciences, Bristol Medical School, University of Bristol, UK
3. Benjamin R Waterhouse: North Bristol NHS Trust, Bristol, UK
4. Andrew D Torrance: Department of Surgery, Sandwell and West Birmingham NHS Trust
5. Sean Strong: Population Health Sciences, Bristol Medical School, University of Bristol, UK and North Bristol NHS Trust, Bristol, UK
6. Dimitrios Siassakos: Population Health Sciences, Bristol Medical School, University of Bristol, UK and North Bristol NHS Trust, Bristol, UK
7. William Seligman: North Bristol NHS Trust, Bristol, UK
8. Leila Rooshenas: Population Health Sciences, Bristol Medical School, University of Bristol, UK
9. Chris Rogers: Clinical Trials and Evaluation Unit, Bristol Trials Centre, Bristol Medical School, University of Bristol, Bristol, UK

For peer review only

10. Lloyd Rickard: University Hospitals Birmingham NHS Foundation Trust, Birmingham, UK
11. Barnaby C Reeves: Clinical Trials and Evaluation Unit, Bristol Trials Centre, Bristol Medical School, University of Bristol, Bristol, UK
12. Anne Pullyblank: North Bristol NHS Trust, Bristol, UK
13. Caroline Pope: Clinical Trials and Evaluation Unit, Bristol Trials Centre, Bristol Medical School, University of Bristol, Bristol, UK
14. Thomas D Pinkney: Academic Department of Surgery, Queen Elizabeth Hospital, University of Birmingham, UK
15. Samir Pathak: University Hospitals Bristol NHS Foundation Trust, Bristol, UK
16. Anwar Owais: University Hospitals Bristol NHS Foundation Trust, Bristol, UK
17. Jamie O'Callaghan: University Hospitals Bristol NHS Foundation Trust, Bristol, UK
18. Stephen O'Brien: North Bristol NHS Trust, Bristol, UK
19. Dmitri Nepogodiev: Academic Department of Surgery, Queen Elizabeth Hospital, University of Birmingham, UK and University Hospitals Birmingham NHS Foundation Trust, Birmingham, UK
20. Khaldoun Nadi: North Bristol NHS Trust, Bristol, UK
21. Charlotte Murkin: Population Health Sciences, Bristol Medical School, University of Bristol, UK and University Hospitals Bristol NHS Foundation Trust, Bristol, UK
22. Tonia Munder: University Hospitals Bristol NHS Foundation Trust, Bristol, UK
23. Tom Milne: North Bristol NHS Trust, Bristol, UK
24. David Messenger: University Hospitals Bristol NHS Foundation Trust, Bristol, UK
25. Christel McMullan: Academic Department of Surgery, Queen Elizabeth Hospital, University of Birmingham, UK
26. Jonathan M Mathers: Institute of Applied Health Research, University of Birmingham, Birmingham, UK
27. Matthew Mason: University Hospitals Bristol NHS Foundation Trust, Bristol, UK
28. Morwena Marshall: University Hospitals Bristol NHS Foundation Trust, Bristol, UK
29. Rhiannon Macefield: Population Health Sciences, Bristol Medical School, University of Bristol, UK
30. Richard Lovegrove: Worcestershire Acute Hospitals NHS Trust, Worcester, UK
31. Robert J Longman: University Hospitals Bristol NHS Foundation Trust, Bristol, UK
32. Jessica Lloyd: North Bristol NHS Trust, Bristol, UK
33. Jeffrey Lim: University Hospitals Bristol NHS Foundation Trust, Bristol, UK
34. Kathryn Lee: University Hospitals Birmingham NHS Foundation Trust, Birmingham, UK
35. Vijay Korwar: North Bristol NHS Trust, Bristol, UK
36. Daniel Hughes: University Hospitals Bristol NHS Foundation Trust, Bristol, UK
37. George Hill: North Bristol NHS Trust, Bristol, UK
38. Rosie Harris: Clinical Trials and Evaluation Unit, Bristol Trials Centre, Bristol Medical School, University of Bristol, Bristol, UK
39. Mohammed Hamdan: University Hospitals Bristol NHS Foundation Trust, Bristol, UK and North Bristol NHS Trust, Bristol, UK

For peer review only

40. Hannah Gould Brown: University Hospitals Bristol NHS Foundation Trust, Bristol, UK
41. Rachael Gooberman-Hill: Musculoskeletal Research Unit, Translational Health Sciences, Bristol Medical School, University of Bristol, UK
42. James Glasbey: University Hospitals Birmingham NHS Foundation Trust, Birmingham, UK
43. Caroline Fryer: University Hospitals Birmingham NHS Foundation Trust, Birmingham, UK
44. Lucy Ellis: Clinical Trials and Evaluation Unit, Bristol Trials Centre, Bristol Medical School, University of Bristol, Bristol, UK
45. Daisy Elliott: Population Health Sciences, Bristol Medical School, University of Bristol, UK
46. Jo C Dumville: Division of Nursing, Midwifery and Social Work, School of Health Sciences, Faculty of Biology, Medicine and Health, University of Manchester, Manchester Academic Health Science Centre, UK
47. Tim Draycott: North Bristol NHS Trust, Bristol, UK
48. Jenny L Donovan: Population Health Sciences, Bristol Medical School, University of Bristol, UK and NIHR Collaboration for Leadership in Applied Health Research are Care West at University Hospitals Bristol NHS Trust, Bristol, UK
49. Simon Davey: Royal United Hospitals Bath NHS Foundation Trust, Bath, UK
50. David Cotton: University Hospitals Bristol NHS Foundation Trust, Bristol, UK
51. Joanna Coast: Population Health Sciences, Bristol Medical School, University of Bristol, UK
52. Madeleine Clout: Clinical Trials and Evaluation Unit, Bristol Trials Centre, Bristol Medical School, University of Bristol, Bristol, UK
53. Melanie J Calvert: Centre for Patient Reported Outcomes Research and NIHR, Birmingham Biomedical Research Centre, Institute of Applied Health Research, Birmingham University Birmingham, UK
54. Benjamin E Byrne: Population Health Sciences, Bristol Medical School, University of Bristol, UK
55. Oliver D Brown: University Hospitals Bristol NHS Foundation Trust, Bristol, UK
56. Jane M Blazeby: Population Health Sciences, Bristol Medical School, University of Bristol, UK and University Hospitals Bristol NHS Foundation Trust, Bristol, UK
57. Natalie S Blencowe: Population Health Sciences, Bristol Medical School, University of Bristol, UK and University Hospitals Bristol NHS Foundation Trust, Bristol, UK
58. Katarzyna D Bera: University of Oxford, Oxford, UK
59. Joanne Bennett: North Bristol NHS Trust, Bristol, UK
60. Richard Bamford: University Hospitals Bristol NHS Foundation Trust, Bristol, UK
61. Danya Bakhbakhi: North Bristol NHS Trust, Bristol, UK
62. Muhammad Atif: University Hospitals Birmingham NHS Foundation Trust, Birmingham, UK
63. Kate Ashton: Clinical Trials and Evaluation Unit, Bristol Trials Centre, Bristol Medical School, University of Bristol, Bristol, UK
64. Elizabeth Armstrong: North Bristol NHS Trust, Bristol, UK
65. Lazaros Andronis: Division of Health Sciences, University of Warwick, UK
66. Piriyankan Ananthavarathan: University Hospitals Birmingham NHS Foundation Trust, Birmingham, UK

For peer review only

**Abstract**

**Objective:** Surgical site infection (SSI) affects up to 25% of primary surgical wounds. Dressing strategies may influence SSI risk. The Bluebelle Study assessed the feasibility of a multi-centre RCT to evaluate the effectiveness and cost-effectiveness of different dressing strategies to reduce SSI in primary surgical wounds.

**Design:** A pilot, factorial randomised controlled trial (RCT).

**Setting:** Five UK hospitals.

**Participants**: Adults undergoing abdominal surgery with a primary surgical wound.

**Interventions**: Participants were randomised to 'simple dressing', 'glue-as-a-dressing' or 'no dressing', and to the time at which the treatment allocation was disclosed to the surgeon (disclosure time, before or after wound closure).

**Primary and secondary outcome measures**: Feasibility outcomes focussed on recruitment, adherence to randomised allocations, reference assessment of SSI and response rates to participant- and observer-completed questionnaires to assess SSI (proposed primary outcome for main trial), wound experience and symptoms, and quality of life (EQ-5D-5L).

**Results:** Between March and November 2016, 1115 patients were screened; 699 (73.4%) were eligible and approached, 415 (59.4%) consented and 394 (35.3%) were randomised (simple dressing=133; glue=129; 'no dressing'=132). Non-adherence to dressing allocation was 2% (3/133), 6% (8/129) and 15% (20/132) respectively. Adherence to disclosure time was 99% and 86% before and after wound closure respectively. The overall rate of SSI (reference assessment) was 18.1% (51/281). Response rates to the WHQ and other questionnaires ranged from >90% at 4 days to 68% at 4-8 weeks.

**Conclusions:** A definitive RCT of dressing strategies including 'no dressing' is feasible. Further work is needed to optimise questionnaire response rates.

For peer review only

**Strengths and limitations of this study**

- This study is novel pilot *factorial* RCT of wound dressing strategies demonstrating for the first time that randomisation to 'no dressing' on a primary surgical wound was acceptable to staff and patients
- Working with research nurse teams and surgical trainee research collaboratives allowed recruitment to be completed on time and target
- Co-ordinating multiple activities within the pilot study was challenging
- Only 67% of participants completing the SSI questionnaire which will need to be addressed in a main trial

For peer review only

## Introduction

Each year there are over 4.5 million hospital admissions for surgery in England alone. [1] The majority result in 'a closed primary wound' and it is common practice to cover these with a dressing. Despite attempts to minimise infection, many develop a surgical site infection (SSI). This is especially a problem in abdominal surgery and high-risk settings where rates of SSI may reach 25%. [2,3] Surgical site infections require antibiotics and multiple dressings, can delay recovery, reduce quality of life, and are expensive for health services.[4,5]

Abdominal surgery carries one of the highest rates of SSI, particularly if the operation involves the colon or rectum. [3,6] Caesarean section is another procedure which carries a high rate of SSI. Possible ways to reduce SSI include modification of pre-, peri- and post-operative factors, which include optimising wound dressing strategies and examining whether dressings are needed at all. A Cochrane review of dressing strategies, which also reviewed evidence when wounds are left uncovered, was performed in 2011 and since updated.[7,8] The initial review found no difference in rates of SSI between wounds covered with different dressings or left uncovered. The update found insufficient evidence to reach a firm conclusion. Most trials included in the review were small and at high or unclear risk of bias. A subsequent Cochrane review of intra-operative methods to reduce SSI commented on the need for more research in this field.[9,10] In 2014, the UK National Institute of Health therefore called for research proposals to address these issues with feasibility and pilot work to establish if a major randomised controlled trial was possible. The Bluebelle study, a programme of research designed to inform the design of a main trial, [11-15] was funded and included a pilot RCT which is reported here. The aim of the pilot RCT was to establish whether it would be feasible to carry out a large definitive RCT to compare the effectiveness and cost-effectiveness of different dressing strategies to reduce SSIs following elective and unplanned surgery with a primary wound.

**Methods**

Study design

A factorial design was used to investigate adherence to the allocated dressing types and the feasibility of randomising after wound closure. Patients were randomised 1:1:1 to dressing type (simple dressing, glue-as-a-dressing and 'no dressing') and 1:1 to the time at which the dressing allocation was disclosed to the surgeon (revealed before or after wound closure details had been entered onto the study database). The full protocol is published elsewhere.[14] The randomisation scheme was stratified by hospital and specialty (abdominal/obstetric surgery). The rationale for randomising to disclosure time as well as to dressing type was the need to understand whether surgeons' knowledge of treatment allocation influences the quality of wound closure (i.e. if allocation to 'no dressing' leads to surgeons taking more care with wound closure). It was intended to use in-theatre wound photography to assess quality of wound closure in relation to timing of disclosure of allocation; however, it soon became apparent that this outcome measure could not be implemented due to multiple governance and logistical challenges. This paper therefore reports the feasibility of conducting the pilot RCT of different dressing strategies and the feasibility of randomising before or after wound closure.

Study setting and population

The study was set in University Hospitals Bristol NHS Foundation Trust (Bristol Royal Infirmary and St Michael's Hospital), North Bristol NHS Trust (Southmead Hospital), University Hospitals Birmingham NHS Foundation Trust (Queen Elizabeth Hospital) and Worcestershire Acute Hospitals NHS Trust. Included were adult participants undergoing abdominal general or obstetric surgery with a skin incision, who were able and willing to provide consent and complete follow-up at 4-8 weeks. Excluded were people who had undergone major surgery within the previous three months, with wounds that a surgeon planned to close with tissue glue, with contra-indications to dressing allocation and prisoners. Surgery and wound closure were carried out according to local practice.

Feasibility outcomes

Primary feasibility outcomes were whether patients were eligible, consented and recruited to the study, and whether they adhered to randomised allocation (Yes/No). Skin transfers were placed next to the wound to encourage adherence to allocated dressing type after leaving the operating theatre (Figure 1). The feasibility of collecting other data (likely to be used in a main trial) and their completeness was investigated for: patient and observer reported questionnaires measuring SSI with the newly validated Wound Healing Questionnaire (WHQ); patient and observer reported questionnaires to assess symptoms and experiences of wounds and dressings; (EQ-5D-5L) preference-based health-related quality of life (Euroqol EQ-5D-5L); wound complications and resource use.[16] A face-to-face wound assessment was carried out at 6 weeks to validate the WHQ.[17] This assessment was used in combination with data collected at discharge to classify each participant has having had an SSI or not.

Additional wound assessments

The feasibility of participants submitting wound photographs taken at home 4-6 weeks after randomisation and uploading them securely to the trial database was assessed. This was planned with a view to investigating whether the occurrence of an SSI can be ascertained reliably from a photograph at this time by a blinded observer.

Sample size

It was calculated that 920 eligible participants would allow a consent rate of 36% (target number randomised = 330) to be estimated with a 95% confidence interval (CI) of 32% to 39%, and a recruitment rate of 60% with 95% CI of 56% to 64%. A consent rate of 36% was proposed because of previous experience recruiting into surgical trials. It was prespecified that, if adherence to dressing type was <70% in any group, it would be concluded that the main trial would not be feasible.

For peer review only

Statistical analyses

Analyses were directed by a pre-specified analysis plan and performed on an intention-to-treat (ITT) basis. Continuous data were summarised as medians and interquartile ranges (IQRs). Categorical data were summarised as numbers and percentages and 95% confidence intervals. Results were described by centre and by specialty as well as overall. The primary analysis took place when follow-up was complete for all recruited participants. All analyses were performed in Stata version 14.0 (StataCorp LP, College Station, Texas).

Understanding adherence and acceptability to treatment allocation

Semi-structured interviews were conducted with patients and staff within 30 days of surgery to understand issues relating to adherence and acceptability of dressing strategies (especially 'no dressing'). The findings have been reported elsewhere.[13]

Patient and Public Involvement

Patients and the public were involved in several stages of this research. The initial idea came from a patient case study. A Bluebelle study PPI group was established including patients and their carers. Members were involved in study design and set up including commenting on patient facing materials. Patient representatives were on the study steering committee and management group and advised on how to approach patients and ideas for blinding study personnel. Extensive pre-trial feasibility work (published) examined the burden of the intervention and time required to participate in the research with qualitative research. The main trial will continue to include patients are all stages of the work.

For peer review only

## Results

Recruitment and participant details

Between March and November 2016, 1115 patients were screened; 699 (73.4%) were eligible and approached; 415 consented to take part; 394 were randomised (Figure 2). The analysis population consisted of 388 participants (790 wounds), i.e. the 394 randomised participants excluding three participants who withdrew and were unhappy for their data to be used, two participants who were allocated to disclosure of dressing allocation after wound closure and whose randomisation in theatre was not completed, and one participant whose surgery was cancelled. Some patients were consented but not randomised after consent because the study ended. Feasibility outcomes by centre are shown in Table 1. Participants were predominantly women (227/388, 58.5%), overweight (median body mass index 28, interquartile range 24.3 to31.6), ASA grade 2 (203/384, 52.9%) and Caucasian 341/374 (91.2%) (Table 2). Most wounds (93.7%) were closed with sutures and approximately three-quarters of participants were prescribed prophylactic antibiotics. There was no indication that these co-interventions were used differentially by group (Table 2).

Adherence to allocated treatment and timing of randomisation

Adherence to treatment allocation was good. More than 97% of participants correctly received the allocated dressing in theatre with adherence after leaving theatre to group allocation remaining high (86%) through to study exit. Adherence to the time at which their surgeons were informed about the treatment allocation was 99% and 86% before and after wound closure respectively. Interviews with staff and patients indicated that skin transfers were acceptable; nobody objected to their use and most nurses viewed them as useful, although some felt they did not personally need to use the transfers as adherence aids.

Follow up data

For peer review only

Face-to-face SSI reference assessments were performed in 80% of participants, among whom the overall SSI rate was 18.1% (Table 3) Response rates for the participant and observer completed measures of SSI (The WHQ) were 256/378 (68%) and 286/377 (76%) respectively at 4-8 weeks (Table 4). Completion of in-hospital questionnaires to assess wound symptoms (WSQ) and experiences (WEQ) was >90% (355/385). Completion of EQ-5D-5L questionnaires during follow up was 269/382 (70%) at 15 days and 242/377 (64%) at 4-8 weeks. Wound complication data (other than SSI) were completed for 326/388 (84%) participants during the post-operative hospital stay and for 315/378 (83%) participants at 4-8 weeks, with similar completion rates for the three groups. Questionnaires documenting resource use during the admission for surgery were generally well completed (details not shown).

**Discussion**

Almost two thirds of eligible patients consented to take part and adherence to allocated dressing type was good immediately after wound closure and during participants' follow-up. Therefore, it is concluded that a main trial of 'simple dressings', 'glue-as-a-dressing' and 'no dressings' is feasible and acceptable to patients and health professionals.  Implementation of the different randomisation schedules (before or after wound closure) was generally successful. Reference SSI assessments were performed well although other follow up assessments of SSI questionnaires were less satisfactory. Completeness of follow-up, however, was not the focus of the pilot study (foci were recruitment and adherence). It is expected that a future trial would combat these challenges using a complementary armamentarium of measures to enhance follow up (reminders, SMS text messages, telephone follow up etc).

Many previous RCTs have examined interventions to reduce SSI, although the quality and conduct of most studies is low and there is a lack strategic feasibility work[7-9]. The Bluebelle study has addressed many of the key issues. Importantly, it demonstrates that a large, rigorous RCT could be done. It is

For peer review only

likely that a main two-group trial would address whether 'no dressings' are non-inferior to a simple dressing in terms of SSI; this is the comparison is of greatest value to the NHS.[15] A main trial with three groups would be more efficient than a separate trial to test the superiority of 'glue-as-a-dressing' to simple dressings. Although basic/simple dressings are inexpensive, they are used in very high volumes. Evidence that a 'no dressing' strategy is non-inferior may result in significant savings for the health service. However, providing this evidence would likely require likely a very large trial (>10,000 participants) to exclude the possibility of a small increase in the SSI rate in the no dressing group compared to the basic dressing group. Such a large trial would require an efficient design with electronic data capture and a well organised multi-disciplinary clinical and academic team including patient partners.

In the Bluebelle pilot RCT there were contributions from surgical trainees as part of surgical research collaboratives. As observed in other studies, these collaboratives helped the trial to recruit to time and target.  Trainees were also involved in the study design (two trainees were grant co-applicants) and led and contributed to sub-studies[18-19]. The involvement of surgical trainees in high quality trials means that they can gain a research apprenticeship. This will equip their consultant practice with skills to engage in establishing evidence and implementing it as the results of trials become available. There were also complexities of working with surgical trainees, relating to the numbers of people involved and occasional confusion over responsibilities. Centres were required to set up additional processes to streamline communication between the teams and trainees. It is recommended that major trials involving trainee collaboratives consider budgeting for additional administrative support to allow coordination of the efforts of the large numbers of people involved.

Although the study recruited to time and target, there were limitations with the response rates to follow up assessments made by post. The logistics of obtaining the data were complex in this pilot study with three assessments being made (a patient-completed SSI assessment; an observer-

completed SSI assessment; and an independent face-to-face reference SSI assessment) and this required two members of staff. In a main trial, a single assessment would be required. It would also aim follow-up processes (scheduling of despatch and generation of questionnaires, etc.) to be largely automated and for assessments to be conducted electronically (manual processes were used in this pilot RCT). It is therefore believed that it is possible to improve the response rate substantially and we have recommended to the funder that a future main trial be required to demonstrate a high response rate in an internal pilot phase.

In summary, this pilot RCT has informed the feasibility, design and likely conduct of a future main trial of different dressing strategies, including 'no dressing'.[15] A future three group trial could jointly address the hypotheses that: (a) 'glue-as-a-dressing' reduces the risk of SSI compared to 'simple dressing' (superiority of glue-as-a-dressing) and (b) 'no dressing' does not increase the risk of SSI (non-inferiority of 'no dressing'). In such a trial it is proposed that the primary outcome should be a combination of information about SSI collected at discharge (as in this study) and SSI ascertained by the patient-reported questionnaire (the WHQ), providing that a better response rate can be obtained and a cut off score on the WHQ can be established to define SSI. A conventional 'reference' SSI assessment would be impracticable as the primary outcome in a main trial because of the high cost of face-to-face assessments. In view of the observed rates of SSI in this pilot RCT and other studies, such a trial will need to be sizable (> 15,000 patients) to confidently exclude true differences in SSI rate. Another issue to consider for a main trial is the best time to disclose dressing allocation (before or after wound closure). It is concluded that the pilot RCT and feasibility work undertaken within the Bluebelle study has been valuable to inform surgical RCT design. This approach is recommended for other clinical questions with challenges in recruitment and outcome assessment before embarking on a main trial.

For peer review only

**Acknowledgements**

The authors acknowledge the contribution from the nurses and administrative staff within the research teams at all participating hospitals. This study was designed and delivered in collaboration with the Clinical Trials and Evaluation Unit (CTEU), a UKCRC registered clinical trials unit which, as part of the Bristol Trials Centre, is in receipt of National Institute for Health Research CTU support funding.

The Bluebelle team would like to acknowledge the contributions of the following to the study; Laura Magill (University of Birmingham Clinical Trials Unit, Birmingham); Katrina Hurley (Bristol Dental Hospital, Bristol); Trudie Young (University of Glamorgan, Glamorgan); Cathy Winter (North Bristol NHS Trust, Bristol); Jo Chambers (University Hospitals Bristol NHS Foundation Trust, Bristol); Katy Chalmers (Department of Population Health Sciences, Bristol Medical School, Bristol); David Hutton (Clinical Trials and Evaluation Unit, Department of Translational Health Sciences, Bristol Medical School, Bristol); Barry Main (Department of Population Health Sciences, Bristol Medical School, Bristol); Helen Talbot (Clinical Trials and Evaluation Unit, Department of Translational Health Sciences, Bristol Medical School, Bristol). We are grateful to the research nurses who supported the study at our participating centres; University Hospitals Bristol NHS Foundation Trust (Rebecca Houlihan, Joanna Nicklin, Louise Flintoff, Jo Chambers, Karen Bobruk), North Bristol NHS Trust (Helen Cheshire, Suriya Kirkpatrick, Louise Solomon, Alice Jarvie, Cathy Winter, Clementine Skilton, Susan Hughes, Michelle Mayer, Jade Knowlden, Mary Alvarez, Sherrie Villis), University Hospitals Birmingham NHS Foundation Trust (Kelly Hollier, Natalie Jackson, Victoria Hardy, David Tyrell, Sharon Garner, Arlo Whitehouse), Worcestershire Acute Hospitals NHS Trust (Caroline Alton, Jessica Thrush, Julie Wollaston).

For peer review only

**Funding statement**

The Bluebelle study was funded by the National Institute for Health Research HTA Programme (project number 12/200/04) and supported by the MRC ConDuCT-II Hub for Trials Methodology Research (Collaboration and innovation for Difficult and Complex randomised controlled Trials In Invasive procedures - MR/K025643/1). JLD and JMB are NIHR Senior Investigators. JMB is partially funded by the NIHR Biomedical Research Centre at University Hospitals Bristol NHS Foundation Trust and University of Bristol. MJC is partly funded by the NIHR Birmingham Biomedical Research Centre and the NIHR Surgical Reconstruction and Microbiology Research Centre at the University Hospitals Birmingham NHS Foundation Trust and the University of Birmingham.

**Disclaimer**

The views and opinions expressed in this paper are those of the authors and do not necessarily reflect those of the MRC, NIHR HTA, NHS or the Department of Health and Social Care.

**Competing Interests**

None declared.

For peer review only

**Table 1 Outcomes related to the feasibility of identifying and recruiting patients**

| | NBT: General surgery | NBT: Obstetric surgery | UHBham: General surgery | UHBris: General surgery | Worc. General surgery | Total |
|---|---|---|---|---|---|---|
| **No. months open*** | **7** | **7** | **9** | **9** | **4** | **36** |
| **No. potentially eligible recorded/month (median, IQR)** | 14 (3.0, 25.0) | 27 (25.0, 48.0) | 71 (57.0, 80.0) | 21 (13.0, 25.0) | 10 (4.5, 13.0) | 142 (57.0, 152.0) |
| **No. potentially eligible recorded by staff** | 96 | 230 | 558 | 196 | 35 | 1115 |
| **Number (%) potentially eligible confirmed eligible** | 90 (93.8) | 205 (89.1) | 469 (84.1) | 154 (78.6) | 34 (97.1) | 952 (85.4) |
| **Number (%) of eligible who were approached** | 87 (96.7) | 126 (61.5) | 317 (67.6) | 136 (88.3) | 33 (97.1) | 699 (73.4) |
| **Number (%) of eligible approached & consented**** | 65 (74.7) | 81 (64.3) | 120 (37.9) | 127 (93.4) | 22 (66.7) | 415 (59.4) |

IQR: interquartile range, NBT: North Bristol NHS Trust, UHBham: University Hospitals Birmingham NHS Foundation Trust, UHBris: University Hospitals Bristol NHS Foundation Trust, WORC: Worcestershire Acute Hospitals NHS Trust,

* nearest whole month, **not all consented patients were finally randomised

For peer review only

**Table 2: Demographics and clinical details of randomised participants by group**

| | Simple dressing n=131 | Glue as-a-dressing n=126 | 'No dressing' n=131 | Total n=388 |
|---|---|---|---|---|
| **Median age in years (IQR)** | 55 (35.9, 65.3) | 48 (32.3, 66.2) | 53 (36.4, 68.2) | 52 (34.7, 66.9) |
| **Female gender (%)** | 80/131 (61.1) | 75/126 (59.5) | 72/131 (55.0) | 227/388 (58.5) |
| **Median BMI (IQR)*** | 28 (24.5, 31.8) | 27 (24.2, 32.0) | 28 (24.6, 31.0) | 28 (24.3, 31.6) |
| **Ethnicity (%) white** | 120/128 (93.8) | 105/119 (88.2) | 116/127 (91.3) | 341/374 (91.2) |
| **Smoking history (%)** | | | | |
| Current smoker | 16/131 (12.2) | 22/125 (17.6) | 22/130 (16.9) | 60/386 (15.5) |
| Ex-smoker >1 month | 53/131 (40.1) | 36/125 (28.8) | 47/130 (36.2) | 136/386 (35.2) |
| **Current steroids, PO/IV/IM (%)** | 15/131 (11.5) | 4/126 (3.2) | 6/131 (4.6) | 25/388 (6.4) |
| **Diabetes, any type (%)** | 11/130 (8.5) | 10/126 (7.9) | 8/130 (6.2) | 29/386 (7.5) |
| **ASA Class (%)** | | | | |
| 1: Healthy, no medical problems | 43/128 (33.6) | 51/125 (40.8) | 40/131 (30.5) | 134/384 (34.9) |
| 2: Mild systemic disease | 72/128 (56.3) | 58/125 (46.4) | 73/131 (55.7) | 203/384 (52.9) |
| 3/4: Severe systemic disease | 13/128 (10.2) | 16/125 (12.8) | 18/131 (13.7) | 47/384 (12.2) |
| **Wound closure (wounds/patients)** | | | | |

For peer review only

| | | | | |
|---|---|---|---|---|
| Sutures | 240/121 (95.3) | 240/117 (95.1) | 229/117 (90.7) | 709/355 (93.7) |
| Clips | 14/10 (9.9) | 13/6 (6.1) | 16/12 (11.5) | 43/28 (9.2) |
| Steri-strips | 20/9 (7.1) | 1/1 (0.8) | 7/5 (3.8) | 28/15 (4.0) |
| Glue (not planned) | 4/2 (2.0) | 2/2 (2.0) | 4/2 (1.9) | 10/6 (2.0) |
| **Total number of wounds** | 278 | 256 | 256 | 790 |
| **Prophylactic antibiotics (%)** | 101/129 (78.3) | 99/126 (78.6) | 96/130 (73.8) | 296/385 (76.9) |
| **Infection risk of surgery (%)**** | | | | |
| Clean | 46/131 (35.1) | 49/126 (38.9) | 44/131 (33.6) | 139/388 (35.8) |
| Clean-contaminated | 81/131 (61.8) | 72/126 (57.1) | 81/131 (61.8) | 234/388 (60.3) |
| Contaminated/Dirty | 4/131 (3.1) | 5/126 (4.0) | 6/131 (4.6) | 15/388 (3.9) |

BMI: body mass index, ASA: American Society of Anaesthesia, PO: per oral, IV: intravenous, IM:intramuscular, IQR: interquartile range. *4 missing data (simple, glue-as-a-dressing, 'no dressing', 2, 1, 1 respectively), elsewhere when a cell denominator is different to the number in a column header, the difference arises because of missing data for that variable. **Classified by type and urgency of surgery.

For peer review only

**Table 3. Potential trial primary outcome by group**

| | Simple dressing n=131 | Glue as-a-dressing n=126 | 'No dressing' n=131 | Total n=388 |
|---|---|---|---|---|
| **SSI (%)*** | | | | |
| 4-8 week reference | | | | |
| None | 80/97 (82.5) | 83/98 (84.7) | 90/107 (84.1) | 253/302 (83.8) |
| Superficial | 14/97 (14.4) | 14/98 (14.3) | 17/107 (15.9) | 45/302 (14.9) |
| Deep | 3/97 (3.1) | 0/98 (0) | 0/107 (0.0) | 3/302 (1.0) |
| Organ space | 0/97 (0.0) | 1/98 (1.0) | 0/107 (0.0) | 1/302 (0.3) |
| Overall | 17/92 (18.5) | 16/90 (17.8) | 18/99 (18.2) | 51/281 (18.1) |

SSI= surgical site infection, IQR = interquartile range

*when the cell denominator is different to number in column header, the difference arises because of missing data for that variable.

For peer review only

**Table 4. Questionnaire response rates for SSI assessments, wound experience and management questionnaires and EQ-5D-5L by group and overall**

| | Simple dressing n=131 (%) | Glue as-a-dressing n=126 (%) | 'No dressing' n=131 (%) | Total n=388 (%) |
|---|---|---|---|---|
| **SSI reference assessment** | 97/127 (76.4) | 98/122 (80.3) | 107/128 (83.6) | 302/377 (80) |
| **Patient reported SSI assessment (WHQ)** | 84/127 (66.1) | 85/122 (69.7) | 87/129 (67.4) | 256/378 (68) |
| **Observer reported SSI assessment (WHQ)** | 93/127 (73.2) | 92/122 (75.4) | 101/128 (78.9) | 286/377 (76) |
| **Wound questionnaires** | | | | |
| Experience | 118/131 (90.1) | 119/125 (95.2) | 118/129 (91.5) | 355/385 (92.2) |
| Management | 118/131 (90.1) | 121/125 (96.8) | 119/129 (92.2) | 358/385 (93.0) |
| **EQ-5D-5L** | | | | |
| Baseline | 128/131 (97.7) | 126/126 (100) | 131/131 (100) | 385/388 (99.2) |
| 15-days | 90/128 (70.3) | 87/125 (69.6) | 92/129 (71.3) | 269/383 (70.4) |
| 4-8 weeks | 84/127 (66.1) | 78/122 (63.9) | 80/128 (62.5) | 242/377 (64.2) |

SSI= surgical site infection, WHQ = Wound healing questionnaire

For peer review only

Figure 1. Example of a skin transfer (modelled by a volunteer) that was applied near to the wound(s) to promote adherence to the dressing allocation.

Figure 2. CONSORT flow diagram of participants in the Bluebelle study

For peer review only

**Author Contributions**

The Bluebelle study group consists of the writing group, co-investigators and collaborators. Full details of individuals contributions are as follows:

Mark Woodward: Bluebelle study co-investigator, contributing experience of not using dressings on surgical wounds in children who have had abdominal surgery. Nicky J Welton: Bluebelle study co-investigator responsible for conducting a value for information analysis about a full-scale trial. Benjamin R Waterhouse: Bluebelle study collaborator, identified and recruited patients and contributed to study delivery in local trust. Andrew D Torrance: Bluebelle study co-investigator. Sean Strong: Bluebelle study collaborator, identified and recruited patients and contributed to study delivery in local trust. Dimitrios Siassakos: Bluebelle study collaborator, responsible for implementation of the study protocol in one centre. William Seligman: Bluebelle study collaborator, identified and recruited patients and contributed to study delivery in local trust. Leila Rooshenas: Bluebelle study co-investigator responsible for design and delivery of qualitative studies in the pilot trial, to inform trial design and test various aspects of feasibility. Chris Rogers: Bluebelle study co-investigator with responsibility for estimating the target sample size and planning the quantitative analyses. Lloyd Rickard: Bluebelle study collaborator, identified and recruited patients and contributed to study delivery in local trust. Barnaby C Reeves: Bluebelle study co-investigator responsible for the design and methods of the Bluebelle feasibility study and member of the writing group for this manuscript. Anne Pullyblank: Bluebelle study co-investigator with responsibility for set up and delivery in one participating centre of the study in general surgery. Caroline Pope: Bluebelle study collaborator, assisted with setting up and managing the trial. Thomas D Pinkney: Bluebelle study co-investigator with responsibility for one participating centre recruiting patients having abdominal surgery and overall study design. Contributed to the development of the WHQ. Samir Pathak: Bluebelle study collaborator, identified and recruited patients and contributed to study delivery in local trust. Anwar Owais: Bluebelle study collaborator, identified and recruited patients

For peer review only

and contributed to study delivery in local trust. Jamie O'Callaghan: Bluebelle study collaborator, identified and recruited patients and contributed to study delivery in local trust. Stephen O'Brien: Bluebelle study collaborator, identified and recruited patients and contributed to study delivery in local trust. Dmitri Nepogodiev: Bluebelle study collaborator, identified and recruited patients and contributed to study delivery in local trust. Khaldoun Nadi: Bluebelle study collaborator, identified and recruited patients and contributed to study delivery in local trust. Charlotte Murkin: Bluebelle study collaborator, identified and recruited patients and contributed to study delivery in local trust. Tonia Munder: Bluebelle study collaborator, conducted patient follow up and contributed to study delivery in local trust. Tom Milne: Bluebelle study collaborator, researched definitions of simple, complex and no dressings under supervision. Contributed to the development of the WHQ. David Messenger: Bluebelle study collaborator, identified and recruited patients and contributed to study delivery in local trust. Christel McMullan: Bluebelle study collaborator, responsible for qualitative data collection /analysis in the pilot trial. Jonathan M Mathers: Bluebelle study co-investigator with responsibility for co-design and delivery of the qualitative studies in the pilot trial. Matthew Mason: Bluebelle study collaborator, identified and recruited patients and contributed to study delivery in local trust. Morwena Marshall: Bluebelle study collaborator, identified and recruited patients and contributed to study delivery in local trust. Rhiannon Macefield: Bluebelle study co-investigator who led the development and validation of the Wound Healing Questionnaire, under supervision from Jane Blazeby and Barnaby Reeves. Richard Lovegrove: Bluebelle study co-investigator with responsibility for one participating centre recruiting patients having abdominal surgery. Robert J Longman: Bluebelle study co-investigator with responsibility for one participating centre recruiting patients having abdominal surgery. Jessica Lloyd: Bluebelle study collaborator, ientified and recruited patients and contributed to study delivery in local trust. Jeffrey Lim: Bluebelle study collaborator, identified and recruited patients and contributed to study delivery in local trust. Kathryn Lee: Bluebelle study collaborator, identified and recruited patients and contributed to study delivery in local trust. Vijay Korwar: Bluebelle study collaborator, identified and recruited patients

For peer review only

and contributed to study delivery in local trust. Daniel Hughes: Bluebelle study collaborator, identified and recruited patients and contributed to study delivery in local trust. George Hill: Bluebelle study collaborator, identified and recruited patients and contributed to study delivery in local trust. Rosie Harris: Bluebelle study collaborator, responsible for preparing the pilot RCT statistical analysis plan. Mohammed Hamdan: Bluebelle study collaborator, identified and recruited patients and contributed to study delivery in local trust. Hannah Gould Brown: Bluebelle study collaborator, identified and recruited patients and contributed to study delivery in local trust. Rachael Gooberman-Hill: Bluebelle study co-investigator with responsibility for patient and public involvement. James Glasbey: Bluebelle study collaborator, identified and recruited patients and contributed to study delivery in local trust. Caroline Fryer: Bluebelle study collaborator, identified and recruited patients and contributed to study delivery in local trust. Lucy Ellis: Bluebelle study collaborator, prepared the first protocol for the pilot trial. Daisy Elliott: Bluebelle study collaborator, led the development of other study outcome measures. Jo C Dumville: Bluebelle study co-investigator, updated the Cochrane review of wound dressings to consider the use of tissue adhesives as a dressing. Tim Draycott: Bluebelle study co-investigator with responsibility for one participating centre recruiting women having caesarean section. Jenny L Donovan: Bluebelle study co-investigator with responsibility for supervising qualitative research. Simon Davey: Bluebelle study collaborator, identified and recruited patients and contributed to study delivery in local trust. David Cotton: Bluebelle study collaborator, identified and recruited patients and contributed to study delivery in local trust. Joanna Coast: Bluebelle study co-investigator with responsibility for supervising health economic aspects of the pilot trial. Madeleine Clout: Bluebelle study collaborator, assisted in managing the pilot trial and member of the writing group for this manuscript. Melanie J Calvert: Bluebelle study co-investigator advising and supporting development of the WHQ and other outcome measures, the feasibility study and pilot trial design. Benjamin E Byrne: Bluebelle study collaborator, helped set up one participating centre including designing training materials and improving study design and processes. Oliver D Brown: Bluebelle study collaborator, identified and

For peer review only

recruited patients and contributed to study delivery in local trust. Jane M Blazeby: Chief investigator responsible for overall conception and the design of the Bluebelle feasibility study. Member of the writing group of this manuscript. Natalie S Blencowe: Bluebelle study co-investigator who led the survey of wound dressings and contributed to the design of the pilot trial, the development of WHQ and other outcome measures. Katarzyna D Bera: Bluebelle study collaborator, identified and recruited patients and contributed to study delivery in local trust. Joanne Bennett: Bluebelle study collaborator, identified and recruited patients and contributed to study delivery in local trust. Richard Bamford: Bluebelle study collaborator, identified and recruited patients and contributed to study delivery in local trust. Danya Bakhbakhi: Bluebelle study collaborator, identified and recruited patients and contributed to study delivery in local trust. Muhammad Atif: Bluebelle study collaborator, identified and recruited patients and contributed to study delivery in local trust. Kate Ashton: Bluebelle study collaborator, managed the pilot trial, including site initiation visits and the second protocol amendment. Elizabeth Armstrong: Bluebelle study collaborator, identified and recruited patients and contributed to study delivery in local trust. Lazaros Andronis: Bluebelle study co-investigator with responsibility for carrying out health economic aspects of the pilot trial. Piriyankan Ananthavarathan: Bluebelle study collaborator, identified and recruited patients and contributed to study delivery in local trust.

For peer review only

## References


1. The Royal College of Surgeons of England. From Theory to Theatre: Overcoming barrier to innovation in surgery. 2011.

2. A report from the NNIS System. National Nosocomial Infections Surveillance (NNIS) System Report, data summary from January 1992 through June 2004. *American Journal of Infection Control*. 2004;**32**(8):470-85.

3. Health Protection Agency. Surveillance of Surgical Site Infections in NHS Hospitals in England, 2011/12 1-32.

4. Coello R, Charlett A, Wilson J, Ward V, Pearson A, Borriello P. Adverse impact of surgical site infections in English hospitals. *J Hosp Infect*. 2005;**60**(2):93-103.

5. Weber WP, Zwahlen M, Reck S, Feder-Mengus C, Misteli H, Rosenthal R, et al. Economic burden of surgical site infections at a European university hospital. *Infect Control Hosp Epidemiol*. 2008;**29**(7):623-9.

6. Blumetti J, Luu M, Sarosi G, Hartless K, McFarlin J, Parker B, et al. Surgical site infections after colorectal surgery: do risk factors vary depending on the type of infection considered? Surgery. 2007;142(5):704-11.

7. Dumville J, Gray T, Walter C, Sharp C, Page T, Macefield R, et al. Dressings for the prevention of surgical site infection (Review). Cochrane Database of Systematic Reviews. 2016;12(CD003091).


For peer review only

8. Dumville JC, Walter CJ, Sharp CA, Page T. Dressings for the prevention of surgical site infection. Cochrane Database Syst Rev. 2011(7):CD003091.

9. Dumville JC, Gray TA, Walter CJ, Sharp CA, Page T, Macefield R, Blencowe N, Milne TK, Reeves BC, Blazeby J. Dressings for the prevention of surgical site infection. Cochrane Database Syst Rev. 2016 Dec 20;12:CD003091. doi: 10.1002/14651858.CD003091.pub4. Review. PMID: 27996083

10. Walter CJ, Dumville JC, Sharp CA, Page T.Systematic review and meta-analysis of wound dressings in the prevention of surgical-site infections in surgical wounds healing by primary intention. *Br J Surg*. 2012 Sep;99(9):1185-94. doi: 10.1002/bjs.8812. PMID:22777875

11. Elliott D; Bluebelle Study Group. Developing outcome measures assessing wound management and patient experience: a mixed methods study. *BMJ Open*. 2017 Nov 26;7(11):e016155. doi: 10.1136/bmjopen-2017-016155. PMID: 29180591

12. Severn and Peninsula Audit and Research Collaborative for Surgeons (SPARCS) and West Midlands Research Collaborative (WMRC) on behalf of the Bluebelle Study Group. Feasibility work to inform the design of a randomized clinical trial of wound dressings in elective and unplanned abdominal surgery. *Br J Surg*. 2016 Nov;103(12):1738-1744. doi: 10.1002/bjs.10274. Epub 2016 Aug 4. PMID: 27488730

13. Rooshenas L; Bluebelle Study Group; Severn and Peninsula Audit and Research Collaborative for Surgeons; West Midlands Research Collaborative. Bluebelle study (phase A): a mixed-methods feasibility study to inform an RCT of surgical wound dressing

strategies. *BMJ Open*. 2016 Sep 22;6(9):e012635. doi: 10.1136/bmjopen-2016-012635. Erratum in: BMJ Open. 2018 Feb 10;8(2):e012635corr1. PMID: 27660321

14. Bluebelle Study Group, Reeves BC, Andronis L, Blazeby JM, Blencowe NS, Calvert M, Coast J, Draycott T, Donovan JL, Gooberman-Hill R, Longman RJ, Magill L, Mathers JM, Pinkney TD, Rogers CA, Rooshenas L, Torrance A, Welton NJ, Woodward M, Ashton K, Bera KD, Clayton GL, Culliford LA, Dumville JC, Elliott D, Ellis L, Gould-Brown H, Macefield RC, McMullan C, Pope C, Siassakos D, Strong S, Talbot H. A mixed-methods feasibility and external pilot study to inform a large pragmatic randomised controlled trial of the effects of surgical wound dressing strategies on surgical site infections (Bluebelle Phase B): study protocol for a randomised controlled trial. *Trials*. 2017 Aug 29;18(1):401. doi: 10.1186/s13063-017-2102-5. PMID: 2885139

15. Reeves BC, Rooshenas L, Macefield RC, Woodward M, Welton NJ, Waterhouse BR, et al. The Bluebelle Study: a feasibility study of three wound dressing strategies in elective and unplanned surgery. *NIHR Journals Library*. 2018. In press.

16. Macefield R; Bluebelle Study Group. Validation of the Bluebelle Wound Healing Questionnaire (WHQ) for assessment of surgical site infection in close primary wounds after hospital discharge. *Br J Surg.* December 2018. doi: 10.1002/bjs.11008.

17. Herman et al. Development and preliminary testing of the new five-level version of EQ-5D (EQ-5D-5L). Qual Life Res 2011; 20(10): 1727:36 https://www.ncbi.nlm.nih.gov/pubmed/21479777

18. Pinkney T, Calvert M, Bartlett D, Gheorghe A, Redman V, Dowswell G, et al. Impact of wound edge protection devices on surgical site infection after laparotomy: multi-centre randomised controlled trial (ROSSINI trial). *BMJ*. 2013;347:f4305.

19. Nepogodiev D, Chapman SJ, Kolias AG, Fitzgerald JE, Lee M, Blencowe NS; National Surgical Research Collaborative. The effect of trainee research collaboratives in the UK. *Lancet Gastroenterol Hepatol*. 2017 Apr;2(4):247-248. doi: 10.1016/S2468-1253(17)30033-X. Epub 2017 Mar 9. PMID: 28404150

For peer review only

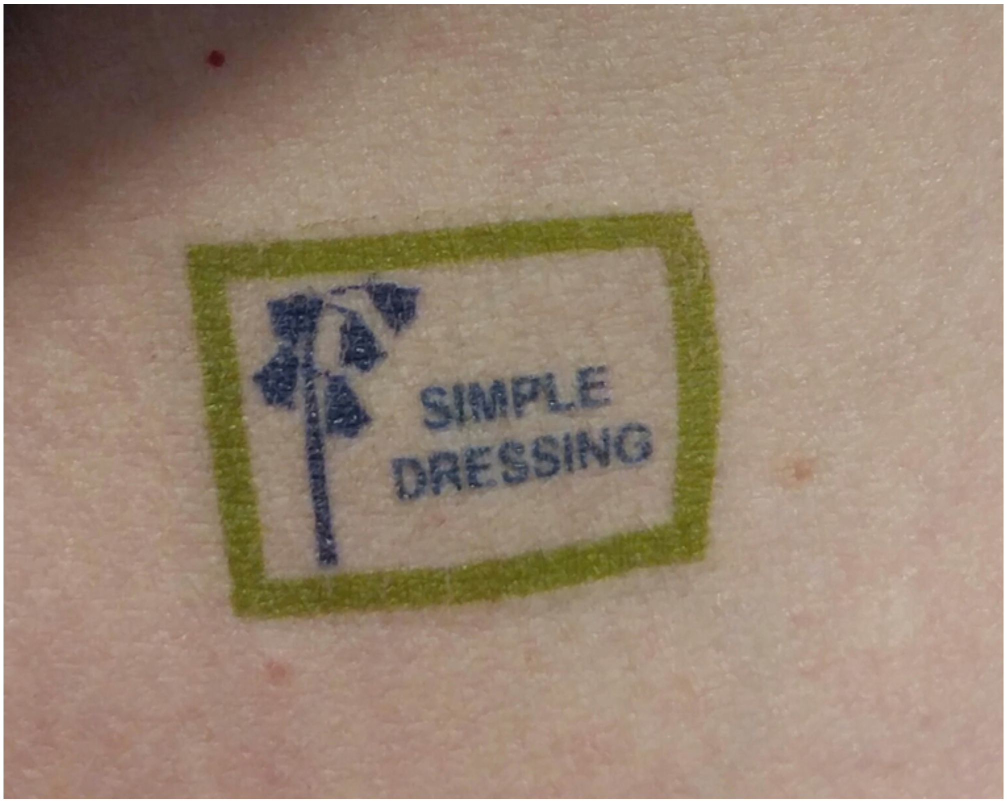


Example of a skin transfer (modelled by a volunteer) that was applied near to the wound(s) to promote adherence to the dressing allocation.

64x51mm (300 x 300 DPI)

**Assessed for eligibility (n=1115)**

**Excluded (n=721)**
Ineligible (N.B may be ineligible for > 1 reason) (n=163)
- Not willing or unable to complete follow-up (n=82)
- Not undergoing primary abdominal or obstetric surgery with a skin incision (n=61)
- Dressing allergy (n=18)
- Previous abdominal surgery or major surgery < 3/12 before (n=6)
- Planned intention to close the wound with glue (n=2)
- Prisoner (n=2)
- Unknown (n=2)
- Age <16 (n=1)

Not approached (n=253)
- In practical to include (n=145)
- Insufficient time to consider the study/urgent surgery (n=74)
- Clinical or surgeon reason (n=11)
- No reason given (n=17)
- Other (n=6)

Did not want to consent (n=284)
- Did not want to be randomised (n=100)
- Personal reasons (n=81)
- Not interested (n=52)
- No reasons given (n=49)
- Other (n=2)

Other (n=21)
- Withdrawn post-consent, pre-randomisation (n=9)
- Not randomised after consent (n=12)

**Randomised (n=394)**

| **Allocated to simple dressing (n=133; before wound closure n=66, after wound closure n=67)** | **Allocated to glue-as-a-dressing (n=129; before wound closure n=64, after wound closure n=65)** | **Allocated to 'no dressing' (n=132; before wound closure n=66, after wound closure n=66)** |
|---|---|---|
| **Withdrawal pre-surgery (n=0)** **Protocol deviations (n=13)** (3 & 10 were non-adherent to dressing allocation & timing of allocation respectively) | **Withdrawal pre-surgery (n=1)** **Protocol deviations (n=17)** (8 & 9 were non-adherent to dressing allocation & timing of allocation respectively) | **Withdrawal pre-surgery (n=0)** **Protocol deviations (n=31)** (20 & 11 were non-adherent to dressing allocation & timing of allocation respectively) |
| **Withdrawal post-surgery (n=6)** | **Withdrawal post-surgery (n=6)** | **Withdrawal post-surgery (n=4)** |
| **Numbers with follow up data** Wound questionnaires* at 4 days (n=118 & 118 respectively) EQ-5D at 15 days & 4-8 weeks (n=90 & n=84 respectively) Observer & participant WHQ at 4-8 wks (n=93 & n=84 respectively) In-hospital & reference SSI | **Numbers with follow up data** Wound questionnaires* at 4 days (n=121 & 119 respectively) EQ-5D at 15 days & 4-8 weeks (n=87 & n=78 respectively) Observer & participant WHQ at 4-8 wks (n=92 & n=85 respectively) In-hospital & reference SSI | **Numbers with follow up data** Wound questionnaires* at 4 days (n=119 & 118 respectively) EQ-5D at 15 days & 4-8 weeks (n=92 & n=80 respectively) Observer & participant WHQ at 4-8 wks (n=101 & n=87 respectively) In-hospital & reference SSI |

Withdrawal pre-surgery as surgery cancelled. Withdrawals post-surgery: participant preference (n=9), death (n=2), randomisation failed in theatre (n=2), clinician chose to withdraw participant (n=2), and one participant required emergency re-operation. WHQ: Wound Healing Questionnaire, SSI: Surgical Site Infection

For peer review only

# CONSORT 2010 checklist of information to include when reporting a pilot or feasibility trial*

| Section/Topic | Item No | Checklist item | Reported on page No |
|---|---|---|---|
| **Title and abstract** | | | |
| | 1a | Identification as a pilot or feasibility randomised trial in the title | |
| | 1b | Structured summary of pilot trial design, methods, results, and conclusions (for specific guidance see CONSORT abstract extension for pilot trials) | |
| **Introduction** | | | |
| Background and objectives | 2a | Scientific background and explanation of rationale for future definitive trial, and reasons for randomised pilot trial | |
| | 2b | Specific objectives or research questions for pilot trial | |
| **Methods** | | | |
| Trial design | 3a | Description of pilot trial design (such as parallel, factorial) including allocation ratio | |
| | 3b | Important changes to methods after pilot trial commencement (such as eligibility criteria), with reasons | |
| Participants | 4a | Eligibility criteria for participants | |
| | 4b | Settings and locations where the data were collected | |
| | 4c | How participants were identified and consented | |
| Interventions | 5 | The interventions for each group with sufficient details to allow replication, including how and when they were actually administered | |
| Outcomes | 6a | Completely defined prespecified assessments or measurements to address each pilot trial objective specified in 2b, including how and when they were assessed | |
| | 6b | Any changes to pilot trial assessments or measurements after the pilot trial commenced, with reasons | |
| | 6c | If applicable, prespecified criteria used to judge whether, or how, to proceed with future definitive trial | |
| Sample size | 7a | Rationale for numbers in the pilot trial | |
| | 7b | When applicable, explanation of any interim analyses and stopping guidelines | |
| Randomisation: | | | |
| Sequence generation | 8a | Method used to generate the random allocation sequence | |
| | 8b | Type of randomisation(s); details of any restriction (such as blocking and block size) | |
| Allocation concealment mechanism | 9 | Mechanism used to implement the random allocation sequence (such as sequentially numbered containers), describing any steps taken to conceal the sequence until interventions were assigned | |

For peer review only

| | | | |
|---|---|---|---|
| Implementation | 10 | Who generated the random allocation sequence, who enrolled participants, and who assigned participants to interventions | |
| Blinding | 11a | If done, who was blinded after assignment to interventions (for example, participants, care providers, those assessing outcomes) and how | |
| | 11b | If relevant, description of the similarity of interventions | |
| Statistical methods | 12 | Methods used to address each pilot trial objective whether qualitative or quantitative | |
| **Results** | | | |
| Participant flow (a diagram is strongly recommended) | 13a | For each group, the numbers of participants who were approached and/or assessed for eligibility, randomly assigned, received intended treatment, and were assessed for each objective | |
| | 13b | For each group, losses and exclusions after randomisation, together with reasons | |
| Recruitment | 14a | Dates defining the periods of recruitment and follow-up | |
| | 14b | Why the pilot trial ended or was stopped | |
| Baseline data | 15 | A table showing baseline demographic and clinical characteristics for each group | |
| Numbers analysed | 16 | For each objective, number of participants (denominator) included in each analysis. If relevant, these numbers should be by randomised group | |
| Outcomes and estimation | 17 | For each objective, results including expressions of uncertainty (such as 95% confidence interval) for any estimates. If relevant, these results should be by randomised group | |
| Ancillary analyses | 18 | Results of any other analyses performed that could be used to inform the future definitive trial | |
| Harms | 19 | All important harms or unintended effects in each group (for specific guidance see CONSORT for harms) | |
| | 19a | If relevant, other important unintended consequences | |
| **Discussion** | | | |
| Limitations | 20 | Pilot trial limitations, addressing sources of potential bias and remaining uncertainty about feasibility | |
| Generalisability | 21 | Generalisability (applicability) of pilot trial methods and findings to future definitive trial and other studies | |
| Interpretation | 22 | Interpretation consistent with pilot trial objectives and findings, balancing potential benefits and harms, and considering other relevant evidence | |
| | 22a | Implications for progression from pilot to future definitive trial, including any proposed amendments | |
| **Other information** | | | |
| Registration | 23 | Registration number for pilot trial and name of trial registry | |
| Protocol | 24 | Where the pilot trial protocol can be accessed, if available | |
| Funding | 25 | Sources of funding and other support (such as supply of drugs), role of funders | |
| | 26 | Ethical approval or approval by research review committee, confirmed with reference number | |

For peer review only

Citation: Eldridge SM, Chan CL, Campbell MJ, Bond CM, Hopewell S, Thabane L, et al. CONSORT 2010 statement: extension to randomised pilot and feasibility trials. BMJ. 2016;355.

*We strongly recommend reading this statement in conjunction with the CONSORT 2010, extension to randomised pilot and feasibility trials, Explanation and Elaboration for important clarifications on all the items. If relevant, we also recommend reading CONSORT extensions for cluster randomised trials, non-inferiority and equivalence trials, non-pharmacological treatments, herbal interventions, and pragmatic trials. Additional extensions are forthcoming: for those and for up to date references relevant to this checklist, see www.consort-statement.org.

For peer review only

BMJ Open

# The Bluebelle pilot randomised controlled trial of three wound dressing strategies to reduce surgical site infection in primary surgical wounds

| | |
|---|---|
| Journal: | *BMJ Open* |
| Manuscript ID | bmjopen-2019-030615.R1 |
| Article Type: | Original research |
| Date Submitted by the Author: | 23-Oct-2019 |
| Complete List of Authors: | Blazeby, Jane; University of Bristol, Department of Population Health Sciences, Bristol Medical School |
| <b>Primary Subject Heading</b>: | Surgery |
| Secondary Subject Heading: | Health services research |
| Keywords: | Randomised controlled trial, Pilot study, Surgical site infection, Wound dressing, Tissue adhesive as a dressing |
| | |

SCHOLARONE™
Manuscripts

# The Bluebelle pilot randomised controlled trial of three wound dressing strategies to reduce surgical site infection in primary surgical wounds

On behalf of the Bluebelle Study Group*

Author for correspondence:

Professor Jane M Blazeby

Centre of Surgical Research,

Bristol Medical School,

University of Bristol,

Canynge Hall,

39 Whatley Road,

Bristol BS8 2PS.

Tel: 0117 9287332

Email: j.m.blazeby@bristol.ac.uk

Word count: 2827

*Authors for 'Bluebelle Study Group':

1. Mark Woodward: University Hospitals Bristol NHS Foundation Trust, Bristol, UK
2. Nicky J Welton: Population Health Sciences, Bristol Medical School, University of Bristol, UK
3. Benjamin R Waterhouse: North Bristol NHS Trust, Bristol, UK
4. Andrew D Torrance: Department of Surgery, Sandwell and West Birmingham NHS Trust
5. Sean Strong: Population Health Sciences, Bristol Medical School, University of Bristol, UK and North Bristol NHS Trust, Bristol, UK
6. Dimitrios Siassakos: Population Health Sciences, Bristol Medical School, University of Bristol, UK and North Bristol NHS Trust, Bristol, UK
7. William Seligman: North Bristol NHS Trust, Bristol, UK
8. Leila Rooshenas: Population Health Sciences, Bristol Medical School, University of Bristol, UK
9. Chris Rogers: Clinical Trials and Evaluation Unit, Bristol Trials Centre, Bristol Medical School, University of Bristol, Bristol, UK



For peer review only - http://bmjopen.bmj.com/site/about/guidelines.xhtml

For peer review only

10. Lloyd Rickard: University Hospitals Birmingham NHS Foundation Trust, Birmingham, UK
11. Barnaby C Reeves: Clinical Trials and Evaluation Unit, Bristol Trials Centre, Bristol Medical School, University of Bristol, Bristol, UK
12. Anne Pullyblank: North Bristol NHS Trust, Bristol, UK
13. Caroline Pope: Clinical Trials and Evaluation Unit, Bristol Trials Centre, Bristol Medical School, University of Bristol, Bristol, UK
14. Thomas D Pinkney: Academic Department of Surgery, Queen Elizabeth Hospital, University of Birmingham, UK
15. Samir Pathak: University Hospitals Bristol NHS Foundation Trust, Bristol, UK
16. Anwar Owais: University Hospitals Bristol NHS Foundation Trust, Bristol, UK
17. Jamie O'Callaghan: University Hospitals Bristol NHS Foundation Trust, Bristol, UK
18. Stephen O'Brien: North Bristol NHS Trust, Bristol, UK
19. Dmitri Nepogodiev: Academic Department of Surgery, Queen Elizabeth Hospital, University of Birmingham, UK and University Hospitals Birmingham NHS Foundation Trust, Birmingham, UK
20. Khaldoun Nadi: North Bristol NHS Trust, Bristol, UK
21. Charlotte Murkin: Population Health Sciences, Bristol Medical School, University of Bristol, UK and University Hospitals Bristol NHS Foundation Trust, Bristol, UK
22. Tonia Munder: University Hospitals Bristol NHS Foundation Trust, Bristol, UK
23. Tom Milne: North Bristol NHS Trust, Bristol, UK
24. David Messenger: University Hospitals Bristol NHS Foundation Trust, Bristol, UK
25. Christel McMullan: Academic Department of Surgery, Queen Elizabeth Hospital, University of Birmingham, UK
26. Jonathan M Mathers: Institute of Applied Health Research, University of Birmingham, Birmingham, UK
27. Matthew Mason: University Hospitals Bristol NHS Foundation Trust, Bristol, UK
28. Morwena Marshall: University Hospitals Bristol NHS Foundation Trust, Bristol, UK
29. Rhiannon Macefield: Population Health Sciences, Bristol Medical School, University of Bristol, UK
30. Richard Lovegrove: Worcestershire Acute Hospitals NHS Trust, Worcester, UK
31. Robert J Longman: University Hospitals Bristol NHS Foundation Trust, Bristol, UK
32. Jessica Lloyd: North Bristol NHS Trust, Bristol, UK
33. Jeffrey Lim: University Hospitals Bristol NHS Foundation Trust, Bristol, UK
34. Kathryn Lee: University Hospitals Birmingham NHS Foundation Trust, Birmingham, UK
35. Vijay Korwar: North Bristol NHS Trust, Bristol, UK
36. Daniel Hughes: University Hospitals Bristol NHS Foundation Trust, Bristol, UK
37. George Hill: North Bristol NHS Trust, Bristol, UK
38. Rosie Harris: Clinical Trials and Evaluation Unit, Bristol Trials Centre, Bristol Medical School, University of Bristol, Bristol, UK
39. Mohammed Hamdan: University Hospitals Bristol NHS Foundation Trust, Bristol, UK and North Bristol NHS Trust, Bristol, UK



For peer review only - http://bmjopen.bmj.com/site/about/guidelines.xhtml

40. Hannah Gould Brown: University Hospitals Bristol NHS Foundation Trust, Bristol, UK
41. Rachael Gooberman-Hill: Musculoskeletal Research Unit, Translational Health Sciences, Bristol Medical School, University of Bristol, UK
42. James Glasbey: University Hospitals Birmingham NHS Foundation Trust, Birmingham, UK
43. Caroline Fryer: University Hospitals Birmingham NHS Foundation Trust, Birmingham, UK
44. Lucy Ellis: Clinical Trials and Evaluation Unit, Bristol Trials Centre, Bristol Medical School, University of Bristol, Bristol, UK
45. Daisy Elliott: Population Health Sciences, Bristol Medical School, University of Bristol, UK
46. Jo C Dumville: Division of Nursing, Midwifery and Social Work, School of Health Sciences, Faculty of Biology, Medicine and Health, University of Manchester, Manchester Academic Health Science Centre, UK
47. Tim Draycott: North Bristol NHS Trust, Bristol, UK
48. Jenny L Donovan: Population Health Sciences, Bristol Medical School, University of Bristol, UK and NIHR Collaboration for Leadership in Applied Health Research are Care West at University Hospitals Bristol NHS Trust, Bristol, UK
49. Simon Davey: Royal United Hospitals Bath NHS Foundation Trust, Bath, UK
50. David Cotton: University Hospitals Bristol NHS Foundation Trust, Bristol, UK
51. Joanna Coast: Population Health Sciences, Bristol Medical School, University of Bristol, UK
52. Madeleine Clout: Clinical Trials and Evaluation Unit, Bristol Trials Centre, Bristol Medical School, University of Bristol, Bristol, UK
53. Melanie J Calvert: Centre for Patient Reported Outcomes Research and NIHR, Birmingham Biomedical Research Centre, Institute of Applied Health Research, Birmingham University Birmingham, UK
54. Benjamin E Byrne: Population Health Sciences, Bristol Medical School, University of Bristol, UK
55. Oliver D Brown: University Hospitals Bristol NHS Foundation Trust, Bristol, UK
56. Jane M Blazeby: Population Health Sciences, Bristol Medical School, University of Bristol, UK and University Hospitals Bristol NHS Foundation Trust, Bristol, UK
57. Natalie S Blencowe: Population Health Sciences, Bristol Medical School, University of Bristol, UK and University Hospitals Bristol NHS Foundation Trust, Bristol, UK
58. Katarzyna D Bera: University of Oxford, Oxford, UK
59. Joanne Bennett: North Bristol NHS Trust, Bristol, UK
60. Richard Bamford: University Hospitals Bristol NHS Foundation Trust, Bristol, UK
61. Danya Bakhbakhi: North Bristol NHS Trust, Bristol, UK
62. Muhammad Atif: University Hospitals Birmingham NHS Foundation Trust, Birmingham, UK
63. Kate Ashton: Clinical Trials and Evaluation Unit, Bristol Trials Centre, Bristol Medical School, University of Bristol, Bristol, UK
64. Elizabeth Armstrong: North Bristol NHS Trust, Bristol, UK
65. Lazaros Andronis: Division of Health Sciences, University of Warwick, UK
66. Piriyankan Ananthavarathan: University Hospitals Birmingham NHS Foundation Trust, Birmingham, UK

For peer review only



For peer review only - http://bmjopen.bmj.com/site/about/guidelines.xhtml

**Abstract**

**Objective:** Surgical site infection (SSI) affects up to 25% of primary surgical wounds. Dressing strategies may influence SSI risk. The Bluebelle Study assessed the feasibility of a multi-centre RCT to evaluate the effectiveness and cost-effectiveness of different dressing strategies to reduce SSI in primary surgical wounds.

**Design:** A pilot, factorial randomised controlled trial (RCT).

**Setting:** Five UK hospitals.

**Participants**: Adults undergoing abdominal surgery with a primary surgical wound.

**Interventions**: Participants were randomised to 'simple dressing', 'glue-as-a-dressing' or 'no dressing', and to the time at which the treatment allocation was disclosed to the surgeon (disclosure time, before or after wound closure).

**Primary and secondary outcome measures**: Feasibility outcomes focussed on recruitment, adherence to randomised allocations, reference assessment of SSI and response rates to participant- and observer-completed questionnaires to assess SSI (proposed primary outcome for main trial), wound experience and symptoms, and quality of life (EQ-5D-5L).

**Results:** Between March and November 2016, 1115 patients were screened; 699 (73.4%) were eligible and approached, 415 (59.4%) consented and 394 (35.3%) were randomised (simple dressing=133; glue=129; 'no dressing'=132). Non-adherence to dressing allocation was 2% (3/133), 6% (8/129) and 15% (20/132) respectively. Adherence to disclosure time was 99% and 86% before and after wound closure respectively. The overall rate of SSI (reference assessment) was 18.1% (51/281). Response rates to the WHQ and other questionnaires ranged from >90% at 4 days to 68% at 4-8 weeks.

**Conclusions:** A definitive RCT of dressing strategies including 'no dressing' is feasible. Further work is needed to optimise questionnaire response rates.



For peer review only - http://bmjopen.bmj.com/site/about/guidelines.xhtml

**Strengths and limitations**

- This pilot factorial RCT addressed whether a main trial of wound dressing strategies was possible.
- The factorial design examined whether intra-operative randomisation was acceptable and feasible.
- Surgical trainee collaboratives and research nurse teams worked together to optimise recruitment.
- Temporary skin transfers adjacent to the surgical wound supported adherence to dressing allocation.
- Follow-up questionnaire response rates were low and need optimisation in a main trial.

For peer review only



For peer review only - http://bmjopen.bmj.com/site/about/guidelines.xhtml

## Introduction

Each year there are over 5 million hospital admissions for surgery in England alone. [1] The majority result in 'a closed primary wound' and it is common practice to cover these with a dressing. Despite attempts to minimise infection, many develop a surgical site infection (SSI). This is especially problematic in abdominal surgery and high-risk settings where rates of SSI may reach 25%. [2,3] Surgical site infections require antibiotics and multiple dressings, can delay recovery, reduce quality of life, and are expensive for health services.[4,5]

Abdominal surgery carries one of the highest rates of SSI, particularly if the operation involves the colon or rectum. [3,6] Caesarean section is another procedure which carries a high rate of SSI.[7] Possible ways to reduce SSI include modification of pre-, peri- and post-operative factors, which include optimising wound dressing strategies and examining whether dressings are needed at all. A Cochrane review of RCTs examining different dressing strategies, which included studies of wounds without a dressing, was performed in 2011 and since updated.[8,9,10] The initial review found no difference in rates of SSI between wounds covered with different dressings or left uncovered. The update found insufficient evidence to reach a firm conclusion. Most trials included in the review were small and at high or unclear risk of bias. A subsequent Cochrane review of intra-operative methods to reduce SSI commented on the need for more research in this field. [,11] In 2014, the UK National Institute of Health Research therefore called for research proposals to address these issues with feasibility and pilot work to establish if a major randomised controlled trial was possible. The Bluebelle study, a programme of research including non-randomised feasibility projects (Phase A) and a pilot RCT (Phase B) was designed to inform the design of a main trial[12-16]. Phase A included interviews with key stakeholders to explore their views of dressings and a trial design[12], a survey of surgical wounds to examine current dressing practice[13] and developmental work to design questionnaires to assess SSI[15] and other aspects of wound management[14]. Here we report the pilot RCT[16].The aim of the pilot RCT was to establish whether it would be feasible to carry out a large



For peer review only - http://bmjopen.bmj.com/site/about/guidelines.xhtml

definitive RCT to compare the effectiveness and cost-effectiveness of different dressing strategies to reduce SSIs following elective and unplanned surgery with a primary wound. Specific objectives were to establish if it was possible to recruit and randomise, to assess the acceptability of, and adherence to, the trial interventions, to examine the feasibility of collecting follow up data, and to establish the measurement properties of the SSI Wound Healing Questionnaire.

For peer review only



For peer review only - http://bmjopen.bmj.com/site/about/guidelines.xhtml

**Methods**

Study design

A factorial design was used to investigate adherence to the allocated dressing types and the feasibility of randomising after wound closure. Patients were randomised 1:1:1 to dressing type (simple dressing, glue-as-a-dressing and 'no dressing') and 1:1 to the time at which the dressing allocation was disclosed to the surgeon (revealed before or after wound closure details had been entered onto the study database). Full details of the interventions are described in the protocol.[16] The randomisation sequences were generated by computer in advance of starting to recruit. Allocation was concealed until participant's eligibility and consent were documented and it was obtained via the internet. Depending on the randomisation result, the dressing allocation was either disclosed immediately, or the user was advised to log back on to the website after the wound had been closed. At the second log on the user was asked to record the timing of wound closure, then the allocation was disclosed. The full protocol is published elsewhere.[16] The randomisation scheme was stratified by hospital and specialty (abdominal/obstetric surgery). The rationale for randomising to disclosure time as well as to dressing type was the need to understand whether surgeons' knowledge of treatment allocation influences the quality of wound closure (i.e. if allocation to 'no dressing' leads to surgeons taking more care with wound closure). It was intended to use in-theatre wound photography to assess quality of wound closure in relation to timing of disclosure of allocation; however, it soon became apparent that this outcome measure could not be implemented due to multiple governance and logistical challenges. This paper therefore reports the feasibility of conducting the pilot RCT of different dressing strategies and the feasibility of randomising before or after wound closure. *Full research ethics approval was obtained from the Frenchay Research Ethics Committee on the 24th February 2015 (REC reference 15/SW/0008) and the trial was registered (International Standardised Randomised Controlled Trial Number 49328913).*

Study setting and population



For peer review only - http://bmjopen.bmj.com/site/about/guidelines.xhtml

The study was set in University Hospitals Bristol NHS Foundation Trust (Bristol Royal Infirmary and St Michael's Hospital), North Bristol NHS Trust (Southmead Hospital), University Hospitals Birmingham NHS Foundation Trust (Queen Elizabeth Hospital) and Worcestershire Acute Hospitals NHS Trust. Included were adult participants undergoing abdominal general or obstetric surgery with a skin incision, who were able and willing to provide consent and complete follow-up at 4-8 weeks. Excluded were people who had undergone major surgery within the previous three months, with wounds that a surgeon planned to close with tissue glue, with contra-indications to dressing allocation and prisoners. Surgery and wound closure were carried out according to local practice.

Feasibility outcomes

Primary feasibility outcomes were whether patients were eligible, consented and recruited to the study, and whether they adhered to randomised allocation (Yes/No). Skin transfers (temporary adherent tattoos) were placed next to the wound to encourage adherence to allocated dressing type after leaving the operating theatre (Figure 1). The feasibility of collecting other data (likely to be used in a main trial) and their completeness was investigated for: patient and observer reported questionnaires measuring SSI with the newly validated Wound Healing Questionnaire (WHQ); patient and observer reported questionnaires to assess symptoms and experiences of wounds and dressings; (EQ-5D-5L) preference-based health-related quality of life (Euroqol EQ-5D-5L) [17]; wound complications and resource use.[18] A face-to-face wound assessment was carried out at 6 weeks to validate the WHQ.[19] This assessment was used in combination with data collected at discharge to classify each participant as having had an SSI or not.

Sample size

It was calculated that 920 eligible participants would allow a consent rate of 36% (target number randomised = 330) to be estimated with a 95% confidence interval (CI) of 32% to 39%, and a



For peer review only - http://bmjopen.bmj.com/site/about/guidelines.xhtml

recruitment rate of 60% with 95% CI of 56% to 64%. A consent rate of 36% was proposed because of previous experience recruiting into surgical trials. It was prespecified that, if adherence to dressing type was <70% in any group, it would be concluded that the main trial would not be feasible.

Statistical analyses

Analyses were directed by a pre-specified analysis plan and performed on an intention-to-treat (ITT) basis. Continuous data were summarised as medians and interquartile ranges (IQRs). Categorical data were summarised as numbers and percentages and 95% confidence intervals. Results were described by centre and by specialty as well as overall. The primary analysis took place when follow-up was complete for all recruited participants. All analyses were performed in Stata version 14.0 (StataCorp LP, College Station, Texas).

Understanding adherence and acceptability to treatment allocation

Semi-structured interviews were conducted with patients and staff within 30 days of surgery to understand issues relating to adherence and acceptability of dressing strategies (especially 'no dressing'). The findings have been reported elsewhere.[12]

Patient and Public Involvement

Patients and the public were involved in several stages of this research. The initial idea came from a patient case study. A Bluebelle study PPI group was established including patients and their carers. Members were involved in study design and set up including commenting on patient facing materials. Patient representatives were on the study steering committee and management group and advised on how to approach patients and ideas for blinding study personnel. Extensive pre-trial feasibility work (published) examined the burden of the intervention and time required to participate in the research with qualitative research. The main trial will continue to include patients throughout all of its stages (design, delivery, analyses, reporting and implementation).



For peer review only - http://bmjopen.bmj.com/site/about/guidelines.xhtml

**Results**

Recruitment and participant details

Between March and November 2016, 1115 patients were screened; 699 (73.4%) were eligible and approached; 415 (37.2%) consented to take part; 394 (35.5%) were randomised (Figure 2). The analysis population consisted of 388 participants (790 wounds), i.e. the 394 randomised participants excluding three participants who withdrew and were unhappy for their data to be used, two participants who were allocated to disclosure of dressing allocation after wound closure and whose randomisation in theatre was not completed, and one participant whose surgery was cancelled. Some patients were consented but not randomised after consent because the study ended. Feasibility outcomes by centre are shown in Table 1. Participants were predominantly women (227/388, 58.5%), overweight (median body mass index 28, interquartile range 24.3 to31.6), ASA grade 2 (203/384, 52.9%) and Caucasian 341/374 (91.2%) (Table 2). Most wounds (93.7%) were closed with sutures and approximately three-quarters of participants were prescribed prophylactic antibiotics. There was no indication that these co-interventions were used differentially by group (Table 2).

Adherence to allocated treatment and timing of randomisation

Adherence to treatment allocation was good. More than 97% of participants correctly received the allocated dressing in theatre with adherence after leaving theatre to group allocation remaining high (86%) through to study exit. Adherence to the time at which their surgeons were informed about the treatment allocation was 99% and 86% before and after wound closure respectively. Interviews with staff and patients indicated that skin transfers were acceptable; nobody objected to their use and most nurses viewed them as useful, although some felt they did not personally need to use the transfers as adherence aids.

Follow up data

For peer review only



For peer review only - http://bmjopen.bmj.com/site/about/guidelines.xhtml

Face-to-face SSI reference assessments were performed in 80% of participants, among whom the overall SSI rate was 18.1% (Table 3) Response rates for the participant and observer completed measures of SSI (The WHQ) were 256/378 (68%) and 286/377 (76%) respectively at 4-8 weeks (Table 4). Completion of in-hospital questionnaires to assess wound symptoms (WSQ) and experiences (WEQ) was >90% (355/385). Completion of EQ-5D-5L questionnaires during follow up was 269/382 (70%) at 15 days and 242/377 (64%) at 4-8 weeks. Wound complication data (other than SSI) were completed for 326/388 (84%) participants during the post-operative hospital stay and for 315/378 (83%) participants at 4-8 weeks, with similar completion rates for the three groups. Questionnaires documenting resource use during the admission for surgery were generally well completed (details not shown).

**Discussion**

Almost two thirds of eligible patients consented to take part and adherence to allocated dressing type was good immediately after wound closure and during participants' follow-up. Therefore, it is concluded that a main trial of 'simple dressings', 'glue-as-a-dressing' and 'no dressings' is feasible and acceptable to patients and health professionals. Implementation of the different randomisation schedules (before or after wound closure) was generally successful. Reference SSI assessments were performed well although other follow up assessments of SSI questionnaires were less satisfactory. Completeness of follow-up, however, was not the focus of the pilot study (foci were recruitment and adherence). It is expected that a future trial would combat these challenges using a complementary armamentarium of measures to enhance follow up (reminders, SMS text messages, telephone follow up etc).

Many previous RCTs have examined interventions to reduce SSI, although the quality and conduct of most studies is low and there is a lack of strategic feasibility work[8-10]. The Bluebelle study has addressed many of the key issues. Importantly, it demonstrates that a large, rigorous RCT could be



For peer review only - http://bmjopen.bmj.com/site/about/guidelines.xhtml

done. In the participating centres there was, however, variation in rates of randomisation (37.9% to 93.4). Some of this variation is likely to be explained by the different approaches used to approaching and screening patients between hospitals. It may also reflect how the study was communicated by individuals at different centres. In a main trial it is expected that training for recruitment and materials used to optimise recruitment will be available based on lessons learnt in this pilot. It is likely that a main two-group trial would address whether 'no dressings' are non-inferior to a simple dressing in terms of SSI; this is the comparison is of greatest value to the NHS.[18] A main trial with three groups would be more efficient than a separate trial to test the superiority of 'glue-as-a-dressing' to simple dressings. Although basic/simple dressings are inexpensive, they are used in very high volumes. Evidence that a 'no dressing' strategy is non-inferior may result in significant savings for the health service. However, providing this evidence would likely require likely a very large trial (>10,000 participants) to exclude the possibility of a small increase in the SSI rate in the no dressing group compared to the basic dressing group. Such a large trial would require an efficient design with electronic data capture and a well organised multi-disciplinary clinical and academic team including patient partners. Since the conception of the Bluebelle study there has been growing use of negative pressure wound therapy on primary wounds to reduce SSI. There is also increasing use of advanced dressings (with interactive properties). Whilst these are of interest to the field, the focus of the proposed main Bluebelle trial is to establish whether 'no dressing' is non-inferior to standard dressings and to gain data to support the use of 'glue-as-a-dressing' on a primary surgical wound.

In the Bluebelle pilot RCT there were contributions from surgical trainees as part of surgical research collaboratives. As observed in other studies, these collaboratives helped the trial to recruit to time and target[-20-21]. Trainees were also involved in the study design (two trainees were grant co-applicants) and led and contributed to sub-studies. The involvement of surgical trainees in high quality trials means that they can gain a research apprenticeship. This will equip their consultant



For peer review only - http://bmjopen.bmj.com/site/about/guidelines.xhtml

practice with skills to engage in establishing evidence and implementing it as the results of trials become available. There were also complexities of working with surgical trainees, relating to the numbers of people involved and occasional confusion over responsibilities. Centres were required to set up additional processes to streamline communication between the teams and trainees. It is recommended that major trials involving trainee collaboratives consider budgeting for additional administrative support to allow coordination of the efforts of the large numbers of people involved.

Although the study recruited to time and target, there were limitations with the response rates to follow up assessments made by post. The logistics of obtaining the data were complex in this pilot study with three assessments being made (a patient-completed SSI assessment; an observer-completed SSI assessment; and an independent face-to-face reference SSI assessment) and this required two members of staff. In a main trial, a single assessment would be required. It would also aim follow-up processes (scheduling of despatch and generation of questionnaires, etc.) to be largely automated and for assessments to be conducted electronically (manual processes were used in this pilot RCT). It is therefore believed that it is possible to improve the response rate substantially and we have recommended to the funder that a future main trial be required to demonstrate a high response rate in an internal pilot phase. In the main trial it may also be possible to supplement questionnaire data about SSIs with wound photography. Further work is on-going exploring this.

In summary, this pilot RCT has informed the feasibility, design and likely conduct of a future main trial of different dressing strategies, including 'no dressing'.[16] A future three group trial could jointly address the hypotheses that: (a) 'glue-as-a-dressing' reduces the risk of SSI compared to 'simple dressing' (superiority of glue-as-a-dressing) and (b) 'no dressing' does not increase the risk of SSI (non-inferiority of 'no dressing'). In such a trial it is proposed that the primary outcome should be a combination of information about SSI collected at discharge (as in this study) and SSI ascertained by the patient-reported questionnaire (the WHQ), providing that a better response rate can be



For peer review only - http://bmjopen.bmj.com/site/about/guidelines.xhtml

obtained and a cut off score on the WHQ can be established to define SSI. A conventional 'reference' SSI assessment would be impracticable as the primary outcome in a main trial because of the high cost of face-to-face assessments. In view of the observed rates of SSI in this pilot RCT and other studies, such a trial will need to be sizable (> 15,000 patients) to confidently exclude true differences in SSI rate. Another issue to consider for a main trial is the best time to disclose dressing allocation (before or after wound closure). It is concluded that the pilot RCT and feasibility work undertaken within the Bluebelle study has been valuable to inform surgical RCT design. This approach is recommended for other clinical questions with challenges in recruitment and outcome assessment before embarking on a main trial.

For peer review only



For peer review only - http://bmjopen.bmj.com/site/about/guidelines.xhtml

**Acknowledgements**

The authors acknowledge the contribution from the nurses and administrative staff within the research teams at all participating hospitals. This study was designed and delivered in collaboration with the Clinical Trials and Evaluation Unit (CTEU), a UKCRC registered clinical trials unit which, as part of the Bristol Trials Centre, is in receipt of National Institute for Health Research CTU support funding.

The Bluebelle team would like to acknowledge the contributions of the following to the study; Laura Magill (University of Birmingham Clinical Trials Unit, Birmingham); Katrina Hurley (Bristol Dental Hospital, Bristol); Trudie Young (University of Glamorgan, Glamorgan); Cathy Winter (North Bristol NHS Trust, Bristol); Jo Chambers (University Hospitals Bristol NHS Foundation Trust, Bristol); Katy Chalmers (Department of Population Health Sciences, Bristol Medical School, Bristol); David Hutton (Clinical Trials and Evaluation Unit, Department of Translational Health Sciences, Bristol Medical School, Bristol); Barry Main (Department of Population Health Sciences, Bristol Medical School, Bristol); Helen Talbot (Clinical Trials and Evaluation Unit, Department of Translational Health Sciences, Bristol Medical School, Bristol). We are grateful to the research nurses who supported the study at our participating centres; University Hospitals Bristol NHS Foundation Trust (Rebecca Houlihan, Joanna Nicklin, Louise Flintoff, Jo Chambers, Karen Bobruk), North Bristol NHS Trust (Helen Cheshire, Suriya Kirkpatrick, Louise Solomon, Alice Jarvie, Cathy Winter, Clementine Skilton, Susan Hughes, Michelle Mayer, Jade Knowlden, Mary Alvarez, Sherrie Villis), University Hospitals Birmingham NHS Foundation Trust (Kelly Hollier, Natalie Jackson, Victoria Hardy, David Tyrell, Sharon Garner, Arlo Whitehouse), Worcestershire Acute Hospitals NHS Trust (Caroline Alton, Jessica Thrush, Julie Wollaston).

For peer review only



For peer review only - http://bmjopen.bmj.com/site/about/guidelines.xhtml

**Funding statement**

The Bluebelle study was funded by the National Institute for Health Research HTA Programme (project number 12/200/04) and supported by the MRC ConDuCT-II Hub for Trials Methodology Research (Collaboration and innovation for Difficult and Complex randomised controlled Trials In Invasive procedures - MR/K025643/1). JLD and JMB are NIHR Senior Investigators. JMB is partially funded by the NIHR Biomedical Research Centre at University Hospitals Bristol NHS Foundation Trust and University of Bristol. MJC is partly funded by the NIHR Birmingham Biomedical Research Centre and the NIHR Surgical Reconstruction and Microbiology Research Centre at the University Hospitals Birmingham NHS Foundation Trust and the University of Birmingham. The funders had no role in the design of the study, the collection and analyses of the data, the interpretation of the findings or in drafting this manuscript

**Disclaimer**

The views and opinions expressed in this paper are those of the authors and do not necessarily reflect those of the MRC, NIHR HTA, NHS or the Department of Health and Social Care.

**Competing Interests**

None declared.

**Data availability statement**

Data are available upon reasonable request. Requests can be made to bluebelle_trial@bristol.ac.uk. Anonymised individual participant data is available for secondary research, conditional on assurance that the proposed use of the data is compliant with the MRC Policy on Data Sharing regarding scientific quality, ethical requirements and value for money.



For peer review only - http://bmjopen.bmj.com/site/about/guidelines.xhtml

**Table 1 Outcomes related to the feasibility of identifying and recruiting patients**

| | NBT: General surgery | NBT: Obstetric surgery | UHBham: General surgery | UHBris: General surgery | Worc: General surgery | Total |
|---|---|---|---|---|---|---|
| **No. months open*** | **7** | **7** | **9** | **9** | **4** | **36** |
| **No. potentially eligible recorded/month (median, IQR)** | 14 (3.0, 25.0) | 27 (25.0, 48.0) | 71 (57.0, 80.0) | 21 (13.0, 25.0) | 10 (4.5, 13.0) | 142 (57.0, 152.0) |
| **No. potentially eligible recorded by staff** | 96 | 230 | 558 | 196 | 35 | 1115 |
| **Number (%) potentially eligible confirmed eligible** | 90 (93.8) | 205 (89.1) | 469 (84.1) | 154 (78.6) | 34 (97.1) | 952 (85.4) |
| **Number (%) of eligible who were approached** | 87 (96.7) | 126 (61.5) | 317 (67.6) | 136 (88.3) | 33 (97.1) | 699 (73.4) |
| **Number (%) of eligible approached & consented**** | 65 (74.7) | 81 (64.3) | 120 (37.9) | 127 (93.4) | 22 (66.7) | 415 (59.4) |

IQR: interquartile range, NBT: North Bristol NHS Trust, UHBham: University Hospitals Birmingham NHS Foundation Trust, UHBris: University Hospitals Bristol NHS Foundation Trust, WORC: Worcestershire Acute Hospitals NHS Trust,

* nearest whole month, **not all consented patients were finally randomised

For peer review only

**Table 2: Demographics and clinical details of randomised participants by group**

| | Simple dressing n=131 | Glue as-a-dressing n=126 | 'No dressing' n=131 | Total n=388 |
|---|---|---|---|---|
| **Median age in years (IQR)** | 55 (35.9, 65.3) | 48 (32.3, 66.2) | 53 (36.4, 68.2) | 52 (34.7, 66.9) |
| **Female gender (%)** | 80/131 (61.1) | 75/126 (59.5) | 72/131 (55.0) | 227/388 (58.5) |
| **Median BMI (IQR)*** | 28 (24.5, 31.8) | 27 (24.2, 32.0) | 28 (24.6, 31.0) | 28 (24.3, 31.6) |
| **Ethnicity (%) white** | 120/128 (93.8) | 105/119 (88.2) | 116/127 (91.3) | 341/374 (91.2) |
| **Smoking history (%)** | | | | |
| Current smoker | 16/131 (12.2) | 22/125 (17.6) | 22/130 (16.9) | 60/386 (15.5) |
| Ex-smoker >1 month | 53/131 (40.1) | 36/125 (28.8) | 47/130 (36.2) | 136/386 (35.2) |
| **Current steroids, PO/IV/IM (%)** | 15/131 (11.5) | 4/126 (3.2) | 6/131 (4.6) | 25/388 (6.4) |
| **Diabetes, any type (%)** | 11/130 (8.5) | 10/126 (7.9) | 8/130 (6.2) | 29/386 (7.5) |
| **ASA Class (%)** | | | | |
| 1: Healthy, no medical problems | 43/128 (33.6) | 51/125 (40.8) | 40/131 (30.5) | 134/384 (34.9) |
| 2: Mild systemic disease | 72/128 (56.3) | 58/125 (46.4) | 73/131 (55.7) | 203/384 (52.9) |
| 3/4: Severe systemic disease | 13/128 (10.2) | 16/125 (12.8) | 18/131 (13.7) | 47/384 (12.2) |
| **Wound closure (wounds/patients)** | | | | |

For peer review only



For peer review only - http://bmjopen.bmj.com/site/about/guidelines.xhtml

| | | | | |
|---|---|---|---|---|
| Sutures | 240/121 (95.3) | 240/117 (95.1) | 229/117 (90.7) | 709/355 (93.7) |
| Clips | 14/10 (9.9) | 13/6 (6.1) | 16/12 (11.5) | 43/28 (9.2) |
| Steri-strips | 20/9 (7.1) | 1/1 (0.8) | 7/5 (3.8) | 28/15 (4.0) |
| Glue (not planned) | 4/2 (2.0) | 2/2 (2.0) | 4/2 (1.9) | 10/6 (2.0) |
| **Total number of wounds** | 278 | 256 | 256 | 790 |
| **Prophylactic antibiotics (%)** | 101/129 (78.3) | 99/126 (78.6) | 96/130 (73.8) | 296/385 (76.9) |
| **Infection risk of surgery (%)**** | | | | |
| Clean | 46/131 (35.1) | 49/126 (38.9) | 44/131 (33.6) | 139/388 (35.8) |
| Clean-contaminated | 81/131 (61.8) | 72/126 (57.1) | 81/131 (61.8) | 234/388 (60.3) |
| Contaminated/Dirty | 4/131 (3.1) | 5/126 (4.0) | 6/131 (4.6) | 15/388 (3.9) |

BMI: body mass index, ASA: American Society of Anaesthesia, PO: per oral, IV: intravenous, IM:intramuscular, IQR: interquartile range. *4 missing data (simple, glue-as-a-dressing, 'no dressing', 2, 1, 1 respectively), elsewhere when a cell denominator is different to the number in a column header, the difference arises because of missing data for that variable. **Classified by type and urgency of surgery.

For peer review only

**Table 3. Potential trial primary outcome by group**

| | Simple dressing n=131 | Glue as-a-dressing n=126 | 'No dressing' n=131 | Total n=388 |
|---|---|---|---|---|
| **SSI (%)*** | | | | |
| 4-8 week reference | | | | |
| None | 80/97 (82.5) | 83/98 (84.7) | 90/107 (84.1) | 253/302 (83.8) |
| Superficial | 14/97 (14.4) | 14/98 (14.3) | 17/107 (15.9) | 45/302 (14.9) |
| Deep | 3/97 (3.1) | 0/98 (0) | 0/107 (0.0) | 3/302 (1.0) |
| Organ space | 0/97 (0.0) | 1/98 (1.0) | 0/107 (0.0) | 1/302 (0.3) |
| Overall | 17/92 (18.5) | 16/90 (17.8) | 18/99 (18.2) | 51/281 (18.1) |

SSI= surgical site infection, IQR = interquartile range

*when the cell denominator is different to number in column header, the difference arises because of missing data for that variable.

For peer review only



For peer review only - http://bmjopen.bmj.com/site/about/guidelines.xhtml

**Table 4. Questionnaire response rates for SSI assessments, wound experience and management questionnaires and EQ-5D-5L by group and overall**

| | Simple dressing n=131 (%) | Glue as-a-dressing n=126 (%) | 'No dressing' n=131 (%) | Total n=388 (%) |
|---|---|---|---|---|
| **SSI reference assessment** | 97/127 (76.4) | 98/122 (80.3) | 107/128 (83.6) | 302/377 (80) |
| **Patient reported SSI assessment (WHQ)** | 84/127 (66.1) | 85/122 (69.7) | 87/129 (67.4) | 256/378 (68) |
| **Observer reported SSI assessment (WHQ)** | 93/127 (73.2) | 92/122 (75.4) | 101/128 (78.9) | 286/377 (76) |
| **Wound questionnaires** | | | | |
| Experience | 118/131 (90.1) | 119/125 (95.2) | 118/129 (91.5) | 355/385 (92.2) |
| Management | 118/131 (90.1) | 121/125 (96.8) | 119/129 (92.2) | 358/385 (93.0) |
| **EQ-5D-5L** | | | | |
| Baseline | 128/131 (97.7) | 126/126 (100) | 131/131 (100) | 385/388 (99.2) |
| 15-days | 90/128 (70.3) | 87/125 (69.6) | 92/129 (71.3) | 269/383 (70.4) |
| 4-8 weeks | 84/127 (66.1) | 78/122 (63.9) | 80/128 (62.5) | 242/377 (64.2) |

SSI= surgical site infection, WHQ = Wound healing questionnaire



For peer review only - http://bmjopen.bmj.com/site/about/guidelines.xhtml

Figure 1. Example of a skin transfer (modelled by a volunteer) that was applied near to the wound(s) to promote adherence to the dressing allocation.

Figure 2. CONSORT flow diagram of participants in the Bluebelle study

For peer review only



For peer review only - http://bmjopen.bmj.com/site/about/guidelines.xhtml

**Author Contributions**

The Bluebelle study group consists of the writing group, co-investigators and collaborators. Full details of individuals contributions are as follows:

Mark Woodward: Bluebelle study co-investigator, contributing experience of not using dressings on surgical wounds in children who have had abdominal surgery. Nicky J Welton: Bluebelle study co-investigator responsible for conducting a value for information analysis about a full-scale trial. Benjamin R Waterhouse: Bluebelle study collaborator, identified and recruited patients and contributed to study delivery in local trust. Andrew D Torrance: Bluebelle study co-investigator. Sean Strong: Bluebelle study collaborator, identified and recruited patients and contributed to study delivery in local trust. Dimitrios Siassakos: Bluebelle study collaborator, responsible for implementation of the study protocol in one centre. William Seligman: Bluebelle study collaborator, identified and recruited patients and contributed to study delivery in local trust. Leila Rooshenas: Bluebelle study co-investigator responsible for design and delivery of qualitative studies in the pilot trial, to inform trial design and test various aspects of feasibility. Chris Rogers: Bluebelle study co-investigator with responsibility for estimating the target sample size and planning the quantitative analyses. Lloyd Rickard: Bluebelle study collaborator, identified and recruited patients and contributed to study delivery in local trust. Barnaby C Reeves: Bluebelle study co-investigator responsible for the design and methods of the Bluebelle feasibility study and member of the writing group for this manuscript. Anne Pullyblank: Bluebelle study co-investigator with responsibility for set up and delivery in one participating centre of the study in general surgery. Caroline Pope: Bluebelle study collaborator, assisted with setting up and managing the trial. Thomas D Pinkney: Bluebelle study co-investigator with responsibility for one participating centre recruiting patients having abdominal surgery and overall study design. Contributed to the development of the WHQ. Samir Pathak: Bluebelle study collaborator, identified and recruited patients and contributed to study delivery in local trust. Anwar Owais: Bluebelle study collaborator, identified and recruited patients



For peer review only - http://bmjopen.bmj.com/site/about/guidelines.xhtml

and contributed to study delivery in local trust. Jamie O'Callaghan: Bluebelle study collaborator, identified and recruited patients and contributed to study delivery in local trust. Stephen O'Brien: Bluebelle study collaborator, identified and recruited patients and contributed to study delivery in local trust. Dmitri Nepogodiev: Bluebelle study collaborator, identified and recruited patients and contributed to study delivery in local trust. Khaldoun Nadi: Bluebelle study collaborator, identified and recruited patients and contributed to study delivery in local trust. Charlotte Murkin: Bluebelle study collaborator, identified and recruited patients and contributed to study delivery in local trust. Tonia Munder: Bluebelle study collaborator, conducted patient follow up and contributed to study delivery in local trust. Tom Milne: Bluebelle study collaborator, researched definitions of simple, complex and no dressings under supervision. Contributed to the development of the WHQ. David Messenger: Bluebelle study collaborator, identified and recruited patients and contributed to study delivery in local trust. Christel McMullan: Bluebelle study collaborator, responsible for qualitative data collection /analysis in the pilot trial. Jonathan M Mathers: Bluebelle study co-investigator with responsibility for co-design and delivery of the qualitative studies in the pilot trial. Matthew Mason: Bluebelle study collaborator, identified and recruited patients and contributed to study delivery in local trust. Morwena Marshall: Bluebelle study collaborator, identified and recruited patients and contributed to study delivery in local trust. Rhiannon Macefield: Bluebelle study co-investigator who led the development and validation of the Wound Healing Questionnaire, under supervision from Jane Blazeby and Barnaby Reeves. Richard Lovegrove: Bluebelle study co-investigator with responsibility for one participating centre recruiting patients having abdominal surgery. Robert J Longman: Bluebelle study co-investigator with responsibility for one participating centre recruiting patients having abdominal surgery. Jessica Lloyd: Bluebelle study collaborator, identified and recruited patients and contributed to study delivery in local trust. Jeffrey Lim: Bluebelle study collaborator, identified and recruited patients and contributed to study delivery in local trust. Kathryn Lee: Bluebelle study collaborator, identified and recruited patients and contributed to study delivery in local trust. Vijay Korwar: Bluebelle study collaborator, identified and recruited patients



For peer review only - http://bmjopen.bmj.com/site/about/guidelines.xhtml

and contributed to study delivery in local trust. Daniel Hughes: Bluebelle study collaborator, identified and recruited patients and contributed to study delivery in local trust. George Hill: Bluebelle study collaborator, identified and recruited patients and contributed to study delivery in local trust. Rosie Harris: Bluebelle study collaborator, responsible for preparing the pilot RCT statistical analysis plan. Mohammed Hamdan: Bluebelle study collaborator, identified and recruited patients and contributed to study delivery in local trust. Hannah Gould Brown: Bluebelle study collaborator, identified and recruited patients and contributed to study delivery in local trust. Rachael Gooberman-Hill: Bluebelle study co-investigator with responsibility for patient and public involvement. James Glasbey: Bluebelle study collaborator, identified and recruited patients and contributed to study delivery in local trust. Caroline Fryer: Bluebelle study collaborator, identified and recruited patients and contributed to study delivery in local trust. Lucy Ellis: Bluebelle study collaborator, prepared the first protocol for the pilot trial. Daisy Elliott: Bluebelle study collaborator, led the development of other study outcome measures. Jo C Dumville: Bluebelle study co-investigator, updated the Cochrane review of wound dressings to consider the use of tissue adhesives as a dressing. Tim Draycott: Bluebelle study co-investigator with responsibility for one participating centre recruiting women having caesarean section. Jenny L Donovan: Bluebelle study co-investigator with responsibility for supervising qualitative research. Simon Davey: Bluebelle study collaborator, identified and recruited patients and contributed to study delivery in local trust. David Cotton: Bluebelle study collaborator, identified and recruited patients and contributed to study delivery in local trust. Joanna Coast: Bluebelle study co-investigator with responsibility for supervising health economic aspects of the pilot trial. Madeleine Clout: Bluebelle study collaborator, assisted in managing the pilot trial and member of the writing group for this manuscript. Melanie J Calvert: Bluebelle study co-investigator advising and supporting development of the WHQ and other outcome measures, the feasibility study and pilot trial design. Benjamin E Byrne: Bluebelle study collaborator, helped set up one participating centre including designing training materials and improving study design and processes. Oliver D Brown: Bluebelle study collaborator, identified and



For peer review only - http://bmjopen.bmj.com/site/about/guidelines.xhtml

recruited patients and contributed to study delivery in local trust. Jane M Blazeby: Chief investigator responsible for overall conception and the design of the Bluebelle feasibility study. Member of the writing group of this manuscript. Natalie S Blencowe: Bluebelle study co-investigator who led the survey of wound dressings and contributed to the design of the pilot trial, the development of WHQ and other outcome measures. Katarzyna D Bera: Bluebelle study collaborator, identified and recruited patients and contributed to study delivery in local trust. Joanne Bennett: Bluebelle study collaborator, identified and recruited patients and contributed to study delivery in local trust. Richard Bamford: Bluebelle study collaborator, identified and recruited patients and contributed to study delivery in local trust. Danya Bakhbakhi: Bluebelle study collaborator, identified and recruited patients and contributed to study delivery in local trust. Muhammad Atif: Bluebelle study collaborator, identified and recruited patients and contributed to study delivery in local trust. Kate Ashton: Bluebelle study collaborator, managed the pilot trial, including site initiation visits and the second protocol amendment. Elizabeth Armstrong: Bluebelle study collaborator, identified and recruited patients and contributed to study delivery in local trust. Lazaros Andronis: Bluebelle study co-investigator with responsibility for carrying out health economic aspects of the pilot trial. Piriyankan Ananthavarathan: Bluebelle study collaborator, identified and recruited patients and contributed to study delivery in local trust.

For peer review only



For peer review only - http://bmjopen.bmj.com/site/about/guidelines.xhtml

**References**


1. Abbott TEF, Fowler AJ, Dobbs TD, Harrison EM, Gillies MA, Pearse RM. Frequency of surgical treatment and related hospital procedures in the UK: a national ecological study using hospital episode statistics. *Br J Anaesth. 2017;**119**(2):249-257. doi: 10.1093/bja/aex137.*
2. A report from the NNIS System. National Nosocomial Infections Surveillance (NNIS) System Report, data summary from January 1992 through June 2004. *American Journal of Infection Control*. 2004;**32**(8):470-85.
3. Health Protection Agency. Surveillance of Surgical Site Infections in NHS Hospitals in England, 2011/12 1-32.
4. Coello R, Charlett A, Wilson J, Ward V, Pearson A, Borriello P. Adverse impact of surgical site infections in English hospitals. *J Hosp Infect*. 2005;**60**(2):93-103.
5. Weber WP, Zwahlen M, Reck S, Feder-Mengus C, Misteli H, Rosenthal R, et al. Economic burden of surgical site infections at a European university hospital. *Infect Control Hosp Epidemiol*. 2008;**29**(7):623-9.
6. Blumetti J, Luu M, Sarosi G, Hartless K, McFarlin J, Parker B, et al. Surgical site infections after colorectal surgery: do risk factors vary depending on the type of infection considered? *Surgery*. 2007;142(5):704-11.
7. A.P. Betran, J. Ye, A.B. Moller, J. Zhang, A.M. Gulmezoglu, M.R. Torloni. The increasing trend in caesarean section rates: global, regional and national estimates: 1990-2014. *PLoS One.* 2016;11(2): e0148343
8. Dumville JC, Walter CJ, Sharp CA, Page T. Dressings for the prevention of surgical site infection. Cochrane Database Syst Rev. 2011(7):CD003091.
9. Dumville JC, Gray TA, Walter CJ, Sharp CA, Page T, Macefield R, Blencowe N, Milne TK, Reeves BC, Blazeby J. Dressings for the prevention of surgical site infection. Cochrane Database Syst Rev. 2016 Dec 20;12:CD003091. doi: 10.1002/14651858.CD003091.pub4. Review. PMID: 27996083


For peer review only



For peer review only - http://bmjopen.bmj.com/site/about/guidelines.xhtml

10. Walter CJ, Dumville JC, Sharp CA, Page T. Systematic review and meta-analysis of wound dressings in the prevention of surgical-site infections in surgical wounds healing by primary intention. *Br J Surg*. 2012 Sep;99(9):1185-94. doi: 10.1002/bjs.8812. PMID:22777875

11. Liu Z1, Dumville JC, Norman G, Westby MJ, Blazeby J, McFarlane E, Welton NJ, O'Connor L, Cawthorne J, George RP, Crosbie EJ, Rithalia AD, Cheng HY. Intraoperative interventions for preventing surgical site infection: an overview of Cochrane Reviews. *Cochrane Database Syst Rev*. 2018(6)2:CD012653.

12. Rooshenas L; Bluebelle Study Group; Severn and Peninsula Audit and Research Collaborative for Surgeons; West Midlands Research Collaborative. Bluebelle study (phase A): a mixed-methods feasibility study to inform an RCT of surgical wound dressing strategies. *BMJ Open*. 2016 Sep 22;6(9):e012635. doi: 10.1136/bmjopen-2016-012635. Erratum in: BMJ Open. 2018 Feb 10;8(2):e012635corr1. PMID: 27660321

13. Severn and Peninsula Audit and Research Collaborative for Surgeons (SPARCS) and West Midlands Research Collaborative (WMRC) on behalf of the Bluebelle Study Group. Feasibility work to inform the design of a randomized clinical trial of wound dressings in elective and unplanned abdominal surgery. *Br J Surg*. 2016 Nov;103(12):1738-1744. doi: 10.1002/bjs.10274. Epub 2016 Aug 4. PMID: 27488730.

14. Elliott D; Bluebelle Study Group. Developing outcome measures assessing wound management and patient experience: a mixed methods study. *BMJ Open*. 2017 Nov 26;7(11):e016155. doi: 10.1136/bmjopen-2017-016155. PMID: 29180591

15. Macefield RC, Reeves BC, Milne TK, Nicholson A, Blencowe NS, Calvert M, Avery KN, Messenger DE, Bamford R, Pinkney TD, Blazeby JM. Development of a single, practical measure of surgical site infection (SSI) for patient report or observer completion. J Infect Prev. 2017 Jul;18(4):170-179. doi: 10.1177/1757177416689724.

16. Bluebelle Study Group, Reeves BC, Andronis L, Blazeby JM, Blencowe NS, Calvert M, Coast J, Draycott T, Donovan JL, Gooberman-Hill R, Longman RJ, Magill L, Mathers JM, Pinkney TD,



For peer review only - http://bmjopen.bmj.com/site/about/guidelines.xhtml

Rogers CA, Rooshenas L, Torrance A, Welton NJ, Woodward M, Ashton K, Bera KD, Clayton GL, Culliford LA, Dumville JC, Elliott D, Ellis L, Gould-Brown H, Macefield RC, McMullan C, Pope C, Siassakos D, Strong S, Talbot H. A mixed-methods feasibility and external pilot study to inform a large pragmatic randomised controlled trial of the effects of surgical wound dressing strategies on surgical site infections (Bluebelle Phase B): study protocol for a randomised controlled trial. *Trials*. 2017 Aug 29;18(1):401. doi: 10.1186/s13063-017-2102-5. PMID: 2885139

17. Herman et al. Development and preliminary testing of the new five-level version of EQ-5D (EQ-5D-5L). Qual Life Res 2011; 20(10): 1727:36 https://www.ncbi.nlm.nih.gov/pubmed/21479777
18. Reeves BC, Rooshenas L, Macefield RC, Woodward M, Welton NJ, Waterhouse BR, et al. Three wound-dressing strategies to reduce surgical site infection after abdominal surgery: the Bluebelle feasibility study and pilot RCT. *Health Technol Assess*. 2019 Aug;23(39):1-166. doi: 10.3310/hta23390.
19. Macefield R; Bluebelle Study Group. Validation of the Bluebelle Wound Healing Questionnaire (WHQ) for assessment of surgical site infection in close primary wounds after hospital discharge. *Br J Surg.* December 2018. doi: 10.1002/bjs.11008.
20. Pinkney T, Calvert M, Bartlett D, Gheorghe A, Redman V, Dowswell G, et al. Impact of wound edge protection devices on surgical site infection after laparotomy: multi-centre randomised controlled trial (ROSSINI trial). *BMJ*. 2013;347:f4305.
21. Nepogodiev D, Chapman SJ, Kolias AG, Fitzgerald JE, Lee M, Blencowe NS; National Surgical Research Collaborative. The effect of trainee research collaboratives in the UK. *Lancet Gastroenterol Hepatol*. 2017 Apr;2(4):247-248. doi: 10.1016/S2468-1253(17)30033-X. Epub 2017 Mar 9. PMID: 28404150



For peer review only - http://bmjopen.bmj.com/site/about/guidelines.xhtml

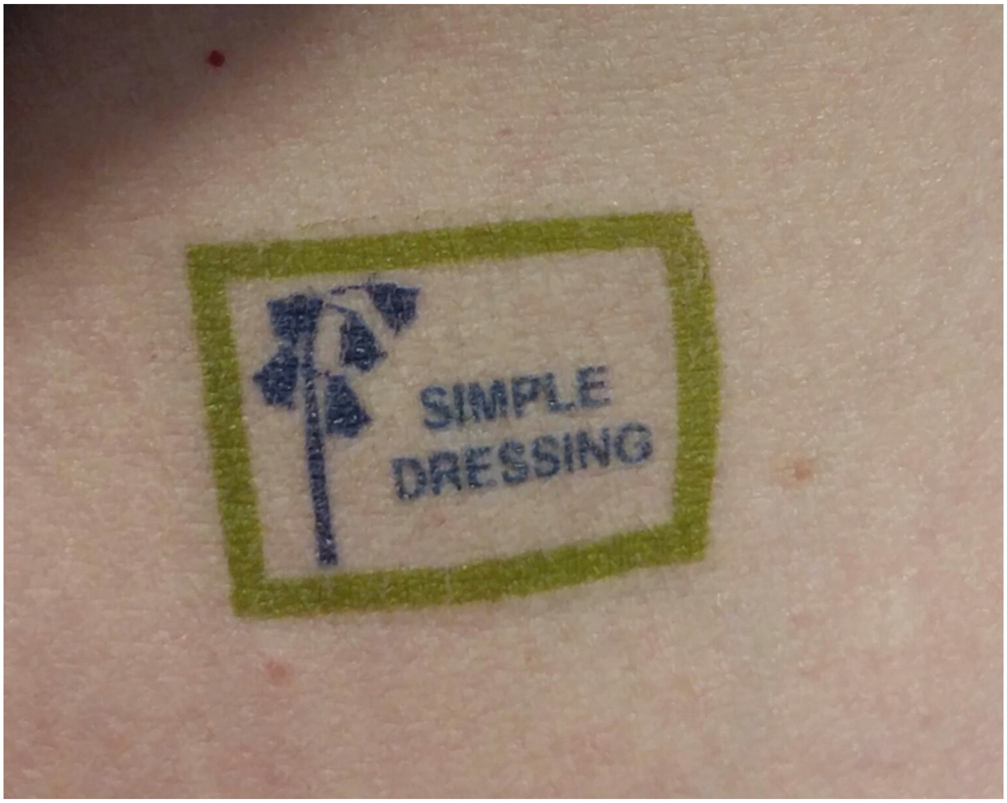


Example of a skin transfer (modelled by a volunteer) that was applied near to the wound(s) to promote adherence to the dressing allocation.

64x51mm (300 x 300 DPI)

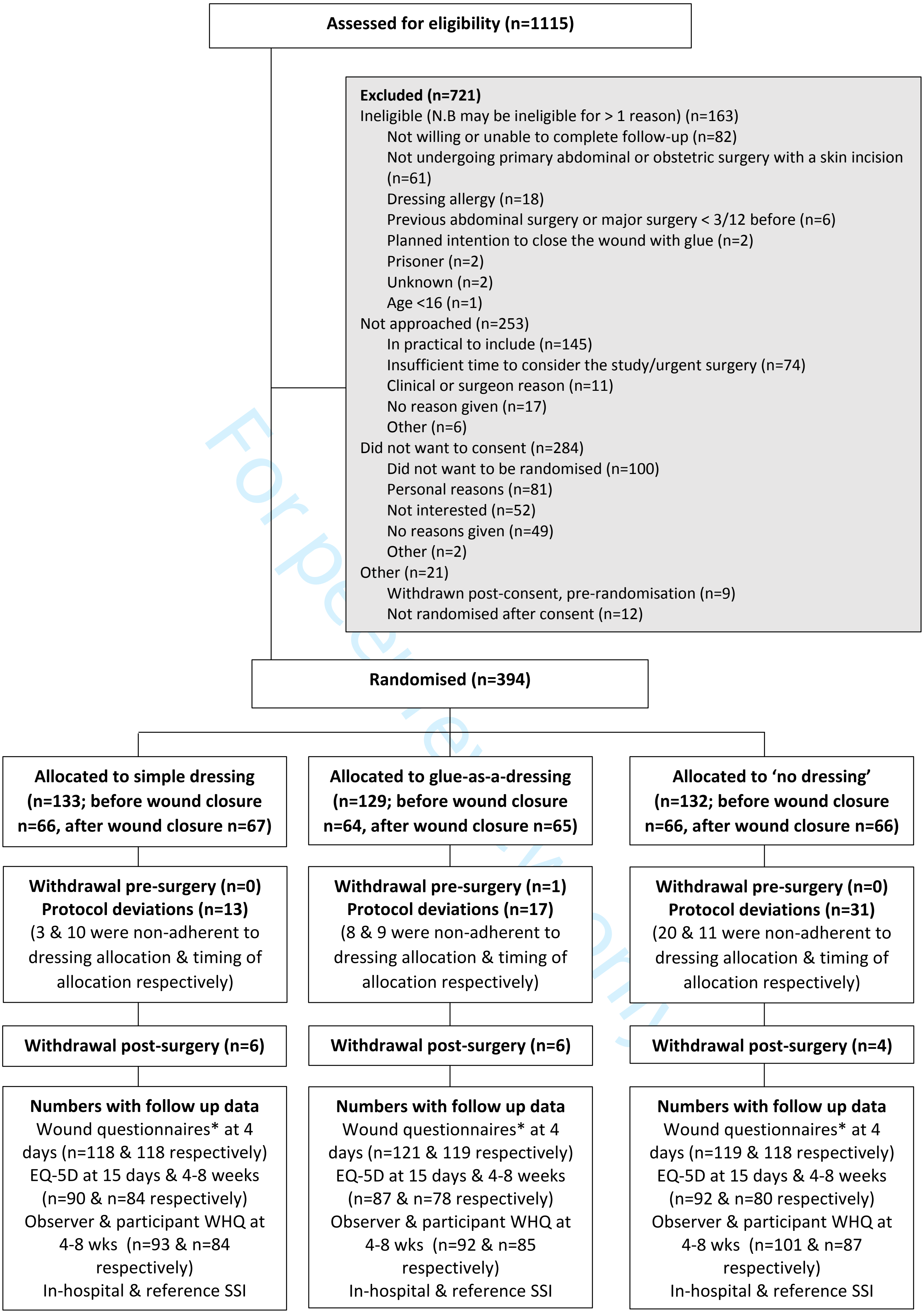


Withdrawal pre-surgery as surgery cancelled. Withdrawals post-surgery: participant preference (n=9), death (n=2), randomisation failed in theatre (n=2), clinician chose to withdraw participant (n=2), and one participant required emergency re-operation. WHQ: Wound Healing Questionnaire, SSI: Surgical Site Infection

# CONSORT 2010 checklist of information to include when reporting a randomised trial*

| Section/Topic | Item No | Checklist item | Reported on page No |
|---|---|---|---|
| **Title and abstract** | | | |
| | 1a | Identification as a randomised trial in the title | 1 |
| | 1b | Structured summary of trial design, methods, results, and conclusions (for specific guidance see CONSORT for abstracts) | 4 |
| **Introduction** | | | |
| Background and objectives | 2a | Scientific background and explanation of rationale | 6 |
| | 2b | Specific objectives or hypotheses | 7 |
| **Methods** | | | |
| Trial design | 3a | Description of trial design (such as parallel, factorial) including allocation ratio | 8 |
| | 3b | Important changes to methods after trial commencement (such as eligibility criteria), with reasons | NA |
| Participants | 4a | Eligibility criteria for participants | 8/9 |
| | 4b | Settings and locations where the data were collected | 9 |
| Interventions | 5 | The interventions for each group with sufficient details to allow replication, including how and when they were actually administered | 8(protocol ref) |
| Outcomes | 6a | Completely defined pre-specified primary and secondary outcome measures, including how and when they were assessed | 9 |
| | 6b | Any changes to trial outcomes after the trial commenced, with reasons | NA |
| Sample size | 7a | How sample size was determined | 10 |
| | 7b | When applicable, explanation of any interim analyses and stopping guidelines | NA |
| Randomisation: | | | |
| Sequence generation | 8a | Method used to generate the random allocation sequence | 8 |
| | 8b | Type of randomisation; details of any restriction (such as blocking and block size) | 8 |
| Allocation concealment mechanism | 9 | Mechanism used to implement the random allocation sequence (such as sequentially numbered containers), describing any steps taken to conceal the sequence until interventions were assigned | 8(protocol ref |
| Implementation | 10 | Who generated the random allocation sequence, who enrolled participants, and who assigned participants to interventions | Protocol ref |
| Blinding | 11a | If done, who was blinded after assignment to interventions (for example, participants, care providers, those | Protocol ref |


For peer review only - http://bmjopen.bmj.com/site/about/guidelines.xhtml

| | | | |
|---|---|---|---|
| | | assessing outcomes) and how | |
| | 11b | If relevant, description of the similarity of interventions | NA |
| Statistical methods | 12a | Statistical methods used to compare groups for primary and secondary outcomes | 10 |
| | 12b | Methods for additional analyses, such as subgroup analyses and adjusted analyses | NA |
| **Results** | | | |
| Participant flow (a diagram is strongly recommended) | 13a | For each group, the numbers of participants who were randomly assigned, received intended treatment, and were analysed for the primary outcome | 12, Figure 2, Table 3 |
| | 13b | For each group, losses and exclusions after randomisation, together with reasons | Figure 2 |
| Recruitment | 14a | Dates defining the periods of recruitment and follow-up | 12 |
| | 14b | Why the trial ended or was stopped | NA |
| Baseline data | 15 | A table showing baseline demographic and clinical characteristics for each group | Table 2 |
| Numbers analysed | 16 | For each group, number of participants (denominator) included in each analysis and whether the analysis was by original assigned groups | Tables 1, 3, 4 |
| Outcomes and estimation | 17a | For each primary and secondary outcome, results for each group, and the estimated effect size and its precision (such as 95% confidence interval) | NA (pilot) |
| | 17b | For binary outcomes, presentation of both absolute and relative effect sizes is recommended | NA (pilot) |
| Ancillary analyses | 18 | Results of any other analyses performed, including subgroup analyses and adjusted analyses, distinguishing pre-specified from exploratory | NA |
| Harms | 19 | All important harms or unintended effects in each group (for specific guidance see CONSORT for harms) | Table 3, effect sizes not estimated |
| **Discussion** | | | |
| Limitations | 20 | Trial limitations, addressing sources of potential bias, imprecision, and, if relevant, multiplicity of analyses | 15 |
| Generalisability | 21 | Generalisability (external validity, applicability) of the trial findings | NA (pilot) |
| Interpretation | 22 | Interpretation consistent with results, balancing benefits and harms, and considering other relevant evidence | 14-16 |
| **Other information** | | | |
| Registration | 23 | Registration number and name of trial registry | 8 |
| Protocol | 24 | Where the full trial protocol can be accessed, if available | Protocol ref 16 |
| Funding | 25 | Sources of funding and other support (such as supply of drugs), role of funders | 18 |

For peer review only

*We strongly recommend reading this statement in conjunction with the CONSORT 2010 Explanation and Elaboration for important clarifications on all the items. If relevant, we also recommend reading CONSORT extensions for cluster randomised trials, non-inferiority and equivalence trials, non-pharmacological treatments, herbal interventions, and pragmatic trials. Additional extensions are forthcoming: for those and for up to date references relevant to this checklist, see www.consort-statement.org.

For peer review only


For peer review only - http://bmjopen.bmj.com/site/about/guidelines.xhtml
